# Critical Design Strategy of Thermogalvanic Hydrogels for Low‐Grade Heat Harvesting

**DOI:** 10.1002/advs.202506038

**Published:** 2025-07-14

**Authors:** Wentao Lin, Shukai Wu, Shuo Niu, Zhe Hu, Guangming Chen, Zhuoxin Liu, Yang Huang, Chao Fang

**Affiliations:** ^1^ Sustainable Energy and Environment Thrust The Hong Kong University of Science and Technology (Guangzhou) Guangzhou Guangdong 511400 China; ^2^ Guangdong Provincial Key Laboratory of New Energy Materials Service Safety College of Materials Science and Engineering Shenzhen University Shenzhen 518055 China; ^3^ Advanced Materials Thrust The Hong Kong University of Science and Technology (Guangzhou) Guangzhou Guangdong 511400 China

**Keywords:** low‐grade heat, material innovation, thermogalvanic hydrogel, thermopower

## Abstract

Low‐grade heat, typically defined as heat at temperatures below 100 °C, is abundant and ubiquitous in the daily environment. However, it is often wasted due to the lack of efficient recovery methods. Thermocells (TECs), which leverage the thermogalvanic effect, provide a promising solution for directly converting low‐grade heat to electricity. Recently, thermogalvanic hydrogels (THs) have emerged as an innovative class of materials for high‐performance TECs due to their giant thermopower, high flexibility, biocompatibility, and low cost. This review comprehensively summarizes the latest advancement in TH research, with a particular focus on the promising design strategies. First, the fundamental mechanisms underlying thermoelectrochemical conversion in THs are systematically scrutinized. Second, the key metrics are outlined for evaluating TECs. Third, current strategies are highlighted for enhancing the thermoelectrochemical performance of THs, including the modifications of polymer matrix, liquid phase, additives, and others. Additionally, the current applications of TH‐based devices are examined in energy harvest and sensing. Finally, the remaining challenges are discussed in the field and provide a forward‐looking perspective on the future development of THs.

## Graphical abstract

This review highlights thermogalvanic hydrogels for efficient low‐grade heat harvesting, with a focus on design strategies. Key aspects include recent advances, thermoelectrochemical conversion mechanisms, performance metrics, enhancement approaches, and current device applications. The discussion concludes with remaining challenges and future perspectives.

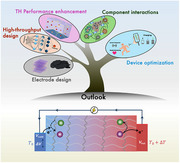

## 1 Introduction

Since the Second Industrial Revolution, humanity has become increasingly dependent on non‐renewable fossil resources, such as coal, oil, and natural gas, to meet the growing demand for electricity. The accelerated consumption of non‐renewable fossil resources for energy production ultimately results in hazardous by‐products and greenhouse gas emissions, resulting in significant environmental damage and pollution.^[^ ^1^, ^2^, ^3^ ^]^ Furthermore, the Paris Agreement mandates that the entire world needs to achieve global net‐zero emissions by 2050 to limit global warming to 1.5 °C, which necessitates a substantial increase in the use of clean renewable energy resources.^[^ ^4^ ^]^ Renewable energy resources, such as wind and solar, have gained popularity and now contribute to over 10% of worldwide electricity generation. They play a crucial role in the transition from primary to renewable energy.^[^ ^5^ ^]^ However, it is important to recognize that regardless of the energy source, the transfer from its original to its end usage also generates various forms of thermal energy, much of which is released into the environment as waste heat but without any practical purpose.

Waste heat is everywhere in our lives. Research by Forman et al. indicates that ≈72% of the global energy output is lost as waste heat, with ≈63% of these waste heat streams originating from sources below 100 °C.^[^ ^6^ ^]^ Consequently, harvesting low‐grade waste heat (typically refers to temperatures below 100 °C) as viable zero‐carbon source of electricity is a highly promising strategy. In 1821, German physicist Thomas Johann Seebeck discovered that various electrical conductors or semiconductors can generate electric potential when subjected to a temperature gradient (Δ*T*). This discovery triggered the development of thermoelectric generators (TEGs), which have been extensively studied and utilized for thermoelectric energy conversion.^[^ ^7^, ^8^, ^9^, ^10^ ^]^ A typical TEG comprises of a p‐type and n‐type semiconductor connected in series. When a Δ*T* is established, the selective thermal movement of mobile charge carriers, including electrons (e^−^) and holes (h^+^), in the material leads to the formation of a potential difference, known as the Seebeck effect. However, TEGs exhibit relatively low efficiency and thermopower at ambient temperatures, rendering them less suitable for effective low‐grade heat harvesting.^[^ ^11^ ^]^

Ionic thermoelectric materials (i‐TMs) are a novel technology that utilizes ions as the charge carriers instead of electrons/holes. A significantly larger thermopower (mV K^−1^ vs µV K^−1^ for TEGs) can be produced because the transport enthalpy of ions is higher than that of electrons.^[^ ^2^, ^12^, ^13^ ^]^ As a result, i‐TMs represents an appealing substitute to traditional electronic thermoelectric materials for heat‐to‐electricity conversion, particularly in the context of harvesting low‐grade heat. Based on i‐TMs, thermal chargeable capacitors (TCCs) have been proposed, which leverage the Soret effect (also known as thermodiffusion (TD) effect) where the differing migration rates of anions and cations under Δ*T* causes their uneven distribution at the hot and cold ends. In TCCs, thermally diffusion ions accumulate on the surface of electrode to create potential difference. However, as these ions cannot pass through, energy is stored under Δ*T* and the output is intermittent when Δ*T* is absent.^[^ ^14^, ^15^ ^]^

When the charge carriers consist of redox couple ions, the thermogalvanic (TG) effect, which is based on thermally driven reversible redox reactions, offers another approach for converting heat into electricity. Under a Δ*T*, redox reactions can occur at the surface of hot/cold electrodes, allowing for the oxidated/reduced ions to transfer continuously through the electrolyte. This process enables thermoelectrochemical cells (TECs) to directly generate a steady current by facilitating the electron exchange at the electrolyte/electrode interface as long as the Δ*T* maintains.^[^ ^16^, ^17^ ^]^ When specific electrodes are employed, the thermopower can also be contributed by the extraction and embedding of metal ions, a phenomenon known as thermoextraction effect.^[^ ^17^, ^18^ ^]^ A more detailed discussion about various mechanisms of ionic thermoelectric effect is provided in Section 2.

The pioneer investigation of ionic thermoelectric electrolyte can be traced back to the studies on ion transport in molten salts.^[^ ^19^, ^20^ ^]^ Subsequent research revealed that ionic solutions based on TG effect could deliver excellent output performance, which has attracted widespread attention.^[^ ^21^ ^]^ However, conventional liquid electrolytes (LEs) suffer from issues such as leakage, complex encapsulation, and integration for practical applications. To mitigate these problems, recent efforts have focused on incorporating quasi solid‐state hydrogel as electrolytes for TECs. From an economic and safety perspective, thermogalvanic hydrogels (THs) are mainly composed of abundant materials of water and polymers, thereby making them cost‐effective and less hazardous compared to those relying on rare, costly, and poisonous elements. Moreover, the thermopower generated by the TH‐based TECs can achieve continuous output, which makes them more efficient than TCCs that typically produce intermittent output.

TH was first proposed in 2016 by Zhou et al.^[^ ^22^ ^]^ The TH was constructed by utilizing polyvinyl alcohol (PVA) as polymer matrix, Fe(CN)_6_^4−/3−^ as redox couple, and water as solvent, which achieved a thermopower of 1.21 mV K^−1^. Recent advancement has significantly enhanced the performance of TH. For instance, the thermopower of TH with Fe(CN)_6_^4−/3−^ redox couple has been increased to 2.02 mV K^−1^ at sub‐zero temperature and can operate continuously below −40 °C by disrupting hydrogen bonds between water molecules.^[^ ^23^ ^]^ In addition, introducing graphene as an additive has been shown to boost the thermopower of TH to 13 mV K^−1^ with the same redox couple.^[^ ^24^ ^]^ Further developments include the application of other redox couples such as Fe^3+^/Fe^2+^, Sn^4+^/Sn^2+^, I‐/I_3_^‒^, and Co(bpy)_3_^3+/2+^, which demonstrated excellent thermoelectrochemical performance.^[^ ^25^, ^26^, ^27^, ^28^, ^29^ ^]^ Besides these thermoelectrochemical improvements, functional TECs based on TH has been explored in applications such as health monitoring and energy supply.^[^ ^22^, ^30^, ^31^, ^32^, ^33^ ^]^

Although THs hold great potential for use in TECs to harvest waste low‐grade heat, the underlying mechanisms for improving thermoelectrochemical performance and critical design strategies still need comprehensive exploration. This review provides a comprehensive overview of current research on TH‐based TECs from the perspectives of mechanisms of heat‐to‐electricity conversion, key parameters of performance evaluation, current studies on the promising design of THs, and emerging applications. Based on these studies, we summarize the challenges and prospects for more efficient TH design, a crucial step toward the advancement of low‐grade heat harvesting technologies.

## 2 Mechanisms of Ionic Heat‐To‐Electricity Conversion

**Figure 1:**
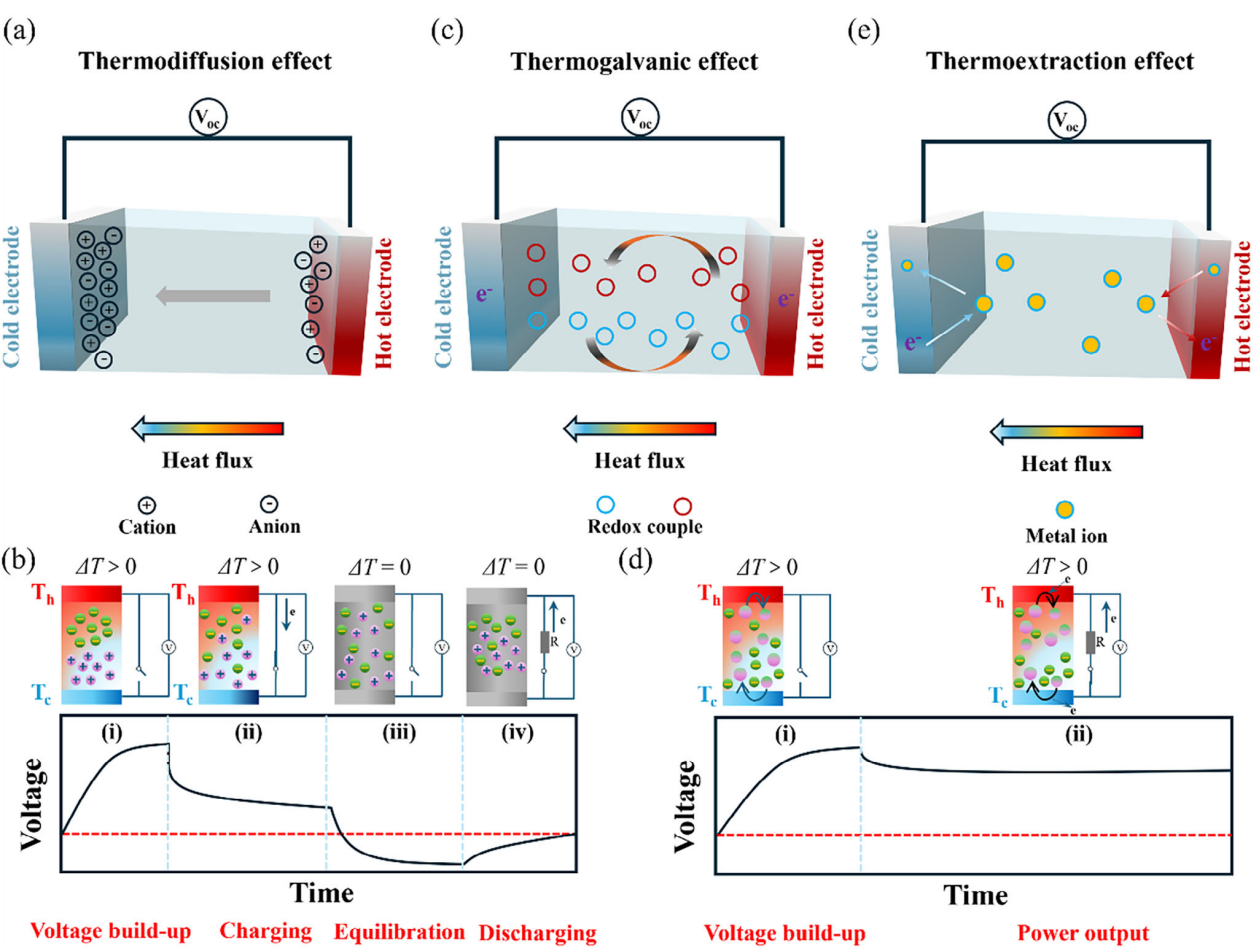
Power generation mechanisms of ionic thermoelectric devices. a) Mechanism of the thermodiffusion effect. b) Four stages for heat‐to‐electricity conversion in TCCs. c) Mechanism of the thermogalvanic effect. d) Two stages for heat‐to‐electricity conversion in TECs. e) Mechanism of the thermoextraction effect.

As mentioned above, i‐TMs can exploit mobile cations or anions to convert heat to electricity. As shown in **Figure** **1**, ionic thermoelectric devices, including TCCs, TECs and thermoextraction cells, typically comprise two electrodes separated by an electrolyte, with the electrodes connected by an external circuit. The presence of a temperature gradient between the electrodes enables these devices to utilize distinct mechanisms for heat‐to‐electricity conversion. This section provides a detailed discussion of heat‐to‐electricity conversion mechanisms in TECs and comparing it with the other two types of mechanisms.

### 2.1 Thermodiffusion Effect

The Soret effect, also known as TD effect, was first proposed by Charles Soret in 1879. It originally described the nonuniform salt concentration in a solution with two ends exhibiting a temperature difference (Δ*T*). The thermodynamic basis of the TD effect is the entropic transfer of ions diffusion between the hot side and cold side, often referred to as the Eastman entropy, and is closely related to the interactions of ions with their surroundings, such as solvents.^[^ ^2^, ^34^, ^35^ ^]^ This unbalanced thermal transport of cations and anions in the electrolyte results in a difference in ion concentration at hot and cold sides. Furthermore, when a Δ*T* is applied, ions are not able to pass through the electrode's interface but accumulate at the ends. Consequently, an electric double layer is formed adjacent to the electrolyte/electrode interface, which generates a potential difference. Currently, researchers have developed various ionic materials based on TD effect including KCl, NaCl, NaOH, Cu_2_SO_4_, ionic liquid, and others.^[^ ^36^, ^37^, ^38^, ^39^, ^40^, ^41^ ^]^ The TCCs leveraging the TD effect have demonstrated capability for energy conversion, thus representing one promising application scenarios based on i‐TMs.

A typical TCC is shown in Figure 1a, the working mode of which is analogous to that of a capacitor. The generated “electricity” cannot be directly transferred to external circuits upon connection. As illustrated in Figure 1b, four‐stage operating steps must be completed to utilize the potential difference generated under a Δ*T*: 1) voltage‐built up, 2) capacitor charging, 3) equilibration, and 4) capacitor discharge. This system is thus more complicated compared to typical TEGs and TECs.^[^ ^37^, ^42^ ^]^ Furthermore, the potential difference diminishes quickly once connected to the external circuit, and a new voltage forms only after disconnecting the external circuit and removing the Δ*T* between two electrodes.^[^ ^15^ ^]^ Therefore, the TCCs are unable to provide continuous and steady output power under a fixed Δ*T*, which limits their practical applications prospects.

### 2.2 Thermogalvanic Effect

The TG effect is the mechanism utilized by TECs for heat‐to‐electricity conversion. As shown in Figure 1c, at least one redox couple must be present in electrolyte for the TG effect to occur. The total redox reaction can be express as:

(1) $$A\Leftrightarrow ne^{-}+B$$

where A and B represent redox couple in different valence state, and *n* is the number of electrons transferred, respectively. The thermodynamics of TG effect involves the temperature‐driven entropy changes during electron transport between the redox couple and the electrode. Under a Δ*T*, the balance of redox reaction was broken, leading to the oxidation of A to B at the hot side and reduction of B to A at the cold side. Once a Δ*T* is applied to two electrodes, the electric potential difference will be generated in successive redox reactions. Consequently, the electrode with high potential acts as anode (oxidation reaction occurs) that supplies electrons to the external circuit, whereas the electrode with low potential acts as cathode (reduction reaction occurs) that receives electrons from the external circuit.

Next, a detailed description of the working process of TECs is provided. Compared to TCCs, the operational process of TECs is simpler, mainly consisting of two steps, as illustrated in Figure 1d. When there is no Δ*T*, the system is in equilibrium, resulting in no electricity generated between the two electrodes due to equal electron energy levels.^[^ ^43^ ^]^ In the first step, the reaction equilibrium is disrupted by the application of Δ*T*, which prompts redox reactions to occur at both electrodes. When the temperature increases at one side while the other side remains unchanged, the hot electrode's electrochemical potential (denoted as *E* (*T*_0_)) rises to *E* (*T*_0_  +  Δ*T*), while the cold electrode's potential remains at *E* (*T*_0_). This difference creates an open circuit voltage (*V_OC_*) between the hot and cold electrodes. In the second step, when connected to an external circuit, it functions like a bridge that allows electrons from the redox reaction to return to the other electrode, resulting in discharge. The transformation of ion valence states at the hot and cold electrodes creates a concentration difference between them. Driven by concentration gradient, the redox ions continuously transfer throughout the electrolyte. Thus, constant electricity supply to the external circuit is facilitated by the exchange of electrons from the redox reactions at the electrodes.

Recently, several extensively reported ionic materials that rely on redox couples have been applied in TECs, including Fe(CN)_6_^4−/3−^, Co (py)_3_^3+/2+^, Fe^3+/2+^, I^−^/I_3_^−^, Cu^2+^/Cu, and Sn^4+^/Sn^2+^.^[^ ^23^, ^43^, ^44^, ^45^, ^46^, ^47^, ^48^ ^]^ Under steady‐state conditions, the thermopower (*S*_i_) can be represented by the slope of a voltage difference (− (*V*_Hot_ − *V*_Cold_)) versus temperature difference (*T*_Hot_ − *T*_Cold_) as:^[^ ^38^ ^]^

(2) $$S_{i}=-\frac{V_{\mathrm{Hot}}-V_{\mathrm{Cold}}}{T_{\mathrm{Hot}}-T_{\mathrm{Cold}}}$$

where *V*_Hot_ is the electrochemical potential of the hot electrode at temperature *T*_Hot_, and *V*_Cold_ is the electrochemical potential of the cold electrode at temperature *T*_Cold_, respectively. Based on the sign of thermopower, it can be classified into p‐type and n‐type. For instance, in the case of aqueous solution, the thermopower of Fe(CN)_6_^4−/3−^ is a typical p‐type redox couple with a value of ≈1.4 mV K^−1^, while I^−^/I_3_^−^ severe as a typical n‐type redox couple with a value of ≈−0.86 mV K^−1^.^[^ ^43^, ^48^ ^]^ To summarize, a comparison between TEGs, TCCs, and TECs is explicitly presented in **Table** **1**.

**Table 1:** Comparison of TEGs, TCCs, and TECs.

	TEGs	TCCs	TECs
Mechanism	Seebeck effect	Thermodiffusion effect	Thermogalvanic effect
Charge Carrier	Holes and electrons	Ions	Redox ions
Key Materials	Solid semiconductors	Liquids/Gels/Polymers	Liquids/Gels
Thermopower	Low	High	Medium
Working mode	Continuous output	Intermittent output	Continuous output
Working temperature	High temperature (e.g., 200–1000 °C)	Low temperature (e.g., <100 °C)	Low temperature (e.g., <100 °C)

### 2.3 Thermoextraction Effect

In addition to the contribution of TD and TG effects to thermopower in electrolytes, the contribution of the thermoextraction effect of electrodes should not be overlooked in specific situations. While the electrode‐involving interaction can be insignificant for traditional noble symmetric metal electrodes, e.g., platinum, it becomes noteworthy when employing carbon‐based (e.g., graphene and carbon nanotube), MXenes, or oxide electrodes (e.g., V_2_O_5_). Generally, the thermoextraction effect contributes to the thermopower contribution through the extraction and embedding of metal ions on the electrode surface.^[^ ^49^, ^50^ ^]^ To be more specific, metal ions are extracted from the hot electrode, then mobilized and migrated to the cold electrode through the electrolyte, followed by charge storage via redox interactions with the electrode. In addition, the extraction of metal ions necessitates the transportation of electrons at the hot electrode. After the extraction of metal ions, the exchange of electrons increases the electrochemical potential at the hot electrode. This results in the formation of a potential difference, as shown in Figure 1e. Since the thermoextraction effect is associated with the electrode and this review mainly focuses on electrolytes, readers are suggested to refer to recent works by Huang et al.,^[^ ^17^ ^]^ Ni et al.,^[^ ^18^ ^]^ and Zhang et al.^[^ ^51^ ^]^ for further details on the thermoextraction effect.

Compared with the TCCs and thermoextraction cells, TECs have the characteristics of being able to maintain continuous and stable power output, rapid mass transfer and charge transfer kinetics at the electrode‐electrolyte interface, and a diverse selection of ion systems, etc. Thanks to this, TECs are being extensively researched to harvest low‐grade heat.

## 3 Key Metrics for Evaluating TECs

TECs convert heat to electricity primarily through TG effect, which involves two essential processes: 1) redox reactions at the hot and cold electrodes, 2) mass transport within the electrolytes. The conversion efficiency and output performance of TECs depend on three interrelated parameters: thermopower (*S*_i_), electrical conductivity (*σ*_i_), and thermal conductivity (*κ*_i_).

### 3.1 Thermopower

Intuitively, *S*_i_ can be defined as ratio of voltage difference to temperature difference, as shown in Equation 2. Additionally, the transformation of the valence state of ions serves as the foundation for TG effect. Thus, *S*_i_ can also be understood through the thermodynamics of this valence state transformation. Under a Δ*T*, the *S*_i_ generated from TG effect is related to the entropy difference (*ΔS*_redox_) of the redox ions and can defined as:^[^ ^52^ ^]^

(3) $$S_{i}=\frac{\left(S_{B}+{\bar{S}}_{B}\right)-\left(S_{A}+{\bar{S}}_{A}\right)-n{\bar{S}}_{e}}{nF}\approx \frac{S_{B}-S_{A}}{nF}=\frac{\Delta S_{\mathrm{redox}}}{nF}$$

where *n* is the number of electrons transferred, *F* is the Faraday's constant, and ${\bar{S}}_{e}$ is transport entropy of electrons in the external circuit. *S*_A_ and *S*_B_ (along with their corresponding ${\bar{S}}_{A}$ and ${\bar{S}}_{B}$) respectively represent the partial molar entropies (Eastman entropies) of *A* and *B*. In most cases, the value of transport entropy of electrons and Eastman entropy was relatively small, and can be neglected.^[^ ^53^ ^]^ Thus, *S*_i_ for TECs is mostly determined by ΔS_redox_ of the redox reaction.

Weaver et al. investigated factors affecting *ΔS*_redox_ and demonstrated that for various low‐spin metal complexes, it can be estimated using the empirical equation:^[^ ^54^ ^]^

(4) $$\Delta S_{\mathrm{redox}}=91.5-2.43AN+86.6\frac{\left(Z_{\mathrm{ox}}^{2}-Z_{\mathrm{re}}^{2}\right)}{r}$$

where *AN* is the solvent acceptor number, $Z_{\mathrm{ox}}$ and $Z_{\mathrm{re}}$ are respectively the charge on the oxidized and reduced species, and *r* is the ionic radius (Å). This equation effectively accounts for solute–solvent interactions and has been successfully applied to correlate thermocouple measurements with solution‐phase dynamics of metal complexes.^[^ ^55^ ^]^ Furthermore, the Born model considers solvents as continuous dielectric medium, and experimentally confirms that ions and their surrounding solvents determine the *ΔS*_redox_.^[^ ^56^, ^57^ ^]^ The Born estimates of *ΔS*_redox_ as follow:

(5) $$\Delta S_{\mathrm{redox}}=-\frac{e^{2}N}{2T\varepsilon }\left(\frac{d\ln\varepsilon }{d\ln T}\right)\left(\frac{Z_{\mathrm{ox}}^{2}}{r_{\mathrm{ox}}}-\frac{Z_{\mathrm{re}}^{2}}{r_{\mathrm{re}}}\right)$$

Here *N* is Avogadro's constant, ε is dielectric constant.

The *S*_i_ can also be determined by the ratio of the equilibrium potential difference between the hot and cold electrodes to the Δ*T*, which is expressed as:

(6) $$\begin{array}{cc} S_{i} & =\frac{R}{nF\Delta T}\left[T_{\mathrm{Hot}}\ln\frac{\gamma_{\mathrm{oH}}^{A}}{\gamma_{\mathrm{rH}}^{B}}+T_{\mathrm{Hot}}\ln\frac{C_{\mathrm{oH}}^{A}}{C_{\mathrm{rH}}^{B}}\right]\\ & \;-\frac{R}{nF\Delta T}\left[T_{\mathrm{Cold}}\ln\frac{\gamma_{\mathrm{oC}}^{A}}{\gamma_{\mathrm{rC}}^{B}}+T_{\mathrm{Cold}}\ln\frac{C_{\mathrm{oC}}^{A}}{C_{\mathrm{rC}}^{B}}\right] \end{array}$$

Here $\gamma_{\mathrm{oH}}^{A},\;\gamma_{\mathrm{rH}}^{B},\;\gamma_{\mathrm{oC}}^{A}$, and $\gamma_{\mathrm{rC}}^{B}$ are activity coefficients of redox ions A/B on the hot/cold electrode, while $C_{\mathrm{oH}}^{A}$, $C_{\mathrm{rH}}^{B}$, $C_{\mathrm{oC}}^{A}$, and $C_{\mathrm{rC}}^{B}$ are concentrations of redox ions A/B on the hot/cold sides, respectively. This equation suggests that the *S*_i_ of TECs mainly depends on the *ΔC*_redox_ of redox ions and *ΔS*_redox_ of the redox reaction. These factors are indicative of the charge density of the redox couple and the interaction with surrounding environment.

### 3.2 Electrical Conductivity and Thermal Conductivity

The performance of TECs is also affected by electrical conductivity *σ*_i_ and thermal conductivity *k*_i_, both of which are regulated by overpotential. During TECs operation, three primary sources contribute to overpotential: 1) Ohmic overpotential, which arises from the resistance of electrolyte, electrode, and circuit. 2) Charge transfer overpotential, which results primarily from the dynamics of electron transfer, that is generated from redox reaction, at the electrodes surface. 3) Mass transfer overpotential, which is mainly related to ion diffusion and convection at the electrode/electrolyte interface and within the bulk electrolyte.

The electrical conductivity (σ_i_) of THs can be calculated by Nyquist plot via the equation:

(7) $$\sigma_{i}=\frac{L}{R_{b}A}$$

where *L* is the thickness of THs, *R*_b_ is the bulk resistance, and *A* is the effective contact area between the TH and the electrodes.

The thermal conductivity (*κ*_i_) of the electrolyte arises from both thermal convection and conduction under a temperature gradient.^[^ ^58^ ^]^ If the *κ*_i_ of the electrolyte is excessively high, it hinders the maintenance of temperature gradient at both ends of the electrode, which can lead to a decrease in the power output during the operation of TECs. Moreover, as the temperatures of both electrodes approach equilibrium, TECs will cease operation. To optimize TECs performance, the *κ*_i_ of electrolyte should be sufficiently low. Currently, THs are considered to meet this requirement due to their low thermal conductivity (<0.5 W m^−1^ K^−1^).^[^ ^59^, ^60^, ^61^ ^]^

The *κ*_i_ of THs can be measured using the transient plane heat source method at room temperature, with calculations performed using a hot disk apparatus. When the probe power remains constant, the time‐dependent temperature rise is described by:^[^ ^62^ ^]^

(8) $$\Delta T_{\mathrm{ave}}\left(\tau \right)=\frac{p_{0}}{\pi^{\frac{3}{2}}r\kappa_{i}}\cdot D\left(\tau \right)$$

where τ is the dimensionless time, Δ*T*_ave_ is the temperature increase on the contact side between the sample and the probe, *p*_0_ is the total power output of the probe, *r* is the radius of the probe disk, *κ*_i_ is the thermal conductivity of the sample, and *D*(τ) is a dimensionless time‐dependent function given by:

(9) $$\tau =\frac{\sqrt{\lambda t}}{\alpha }$$

where *t* is the measurement time from the start of the transient record, λ is the thermal diffusivity of the sample, α is the resistance temperature coefficient. The effective thermal conductivity *κ*_i_ can be calculated based on the slope of line determined by $p_{0}{\left(\pi^{\frac{3}{2}}r\kappa_{i}\right)}^{-1}$ from the temperature increases Δ*T*_ave_ versus the dimensionless time *D*(τ). If the *κ*_i_ of the electrolyte is excessively high, maintaining a temperature gradient at both ends of the electrode becomes challenging, resulting in a decrease during TECs operation. Moreover, as temperatures at both electrodes gradually approach equilibrium, TECs will become inoperative. To optimize TECs performance, it is crucial that the *κ*_i_ of electrolyte is kept sufficiently small.

### 3.3 Cell Performance

The performance of conventional electronic thermoelectric materials can be determined using the dimensionless quality parameter,^[^ ^63^ ^]^ the figure of merit (ZT). It is defined by:

(10) $$ZT=\frac{\sigma S_{i}^{2}}{\kappa_{i}}T$$

where *S*_i_, *T*, σ, and κ_i_ represent the Seebeck coefficient, absolute temperature, electrical conductivity, and thermal conductivity, respectively. However, it may not be entirely suitable for evaluating the thermoelectrochemical performance of ionic thermogalvanic materials. To address this, Yang et al.^[^ ^64^ ^]^ proposed the thermoelectric factor (*Z*_TG_) to better evaluate the performance for TECs. This factor considers ion transport kinetics and electrochemical processes, and is described by the following formula:

(11) $$Z_{\mathrm{TG}}=\frac{S_{\mathrm{TG}}^{2}}{R_{s}K}$$

where *K* is total thermal conductance, *R*_s_ is overall resistance, and *S*_TG_ is thermopower. Furthermore, the temperature‐insensitive maximum power density (*P*_max_/(Δ*T*)^2^) and Carnot‐relative efficiency (η_r_) are two additional parameters for evaluating TEC's performance. The former focuses on output performance, while the latter emphasizes energy conversion efficiency. TCCs, despite their giant thermopower, are limited by a low η_r_ of ≈10^−2^−10^−1^%.^[^ ^64^ ^]^ This low efficiency is mainly attributed to their long thermal charging time, high internal resistance, and the rapid decline of output currents. In contrast, TECs performance better as they can directly and continuously convert heat to electricity. Recent advancements have led to substantially higher η_r_ of TH‐based TECs than TCCs. Notably, an η_r_ close to 5% have been achieved,^[^ ^65^, ^66^ ^]^ thus unlocking the potential of commercialization.^[^ ^67^ ^]^ Equations 12 and 13 show the relationship between the two parameters and previously mentioned interdependent parameters, including *S*_i_, σ_i_, and κ_i_.

(12) $$\frac{P_{\max}}{\Delta T^{2}}=0.25\cdot S_{i}^{2}\cdot \sigma_{i}\cdot \frac{A}{d}$$

(13) $$\eta_{r}=\frac{0.25\cdot S_{i}^{2}\cdot \sigma_{i}}{k_{i}}\cdot T_{h}$$

Here *A*, *d*, and *T*_h_ are the cross‐sectional area of the cell, the thickness of electrolyte, and the temperature at the hot electrode, respectively. The term $S_{i}^{2}\;\times \;\sigma_{i}$ in Equations 12 and 13 can be interpreted as the thermoelectric power factor (PF).^[^ ^68^ ^]^ In summary, in order to provide high thermoelectrochemical performance, it is necessary to maximize *S*_i_ and σ_i_ values while maintaining a low κ_i_. The THs has the potential to meet the above requirements due to their advantageous properties such as satisfactory σ_i_ (i.e., comparable to their liquid counterparts), high *S*_i_ and low κ_i_, thereby meriting further investigations.

## 4 Current Studies on the Critical Design of THs

In practical applications, the use of THs as the electrolyte for TECs has garnered extensive attention. The THs offer advantages such as straightforward manufacturing and effortless integration into devices. They also possess adaptable mechanical properties including elasticity and toughness. By allowing polymer chains to interact with redox couple ions, THs can be engineered for enhanced thermoelectrochemical performance, making them particularly suitable for devices with low‐grade heat harvesting capability. Moreover, THs present various extra functionalities, including stretchability and robustness, freezing tolerance, and self‐healing capability, which further enhance their appeal. In Section 4, we summarize the most promising design strategies for optimizing THs in the context of harvesting low‐grade heat.

### 4.1 Impacts of Polymer Matrix

Hydrogels are polymeric materials characterized by 3D network architectures formed through physical entanglement or chemical cross‐linking of polymers in solution. They have the ability to retain a substantial amount of water or other fluids within their structure.^[^ ^69^ ^]^ Numerous polymers from both synthetic and natural sources have been employed as host matrices for THs, including gelatin, cellulose, PVA, polyacrylamide (PAAm), poly(vinylidene fluoride‐co‐hexafluoropropene) (PVDF‐HFP). The multiple functional groups on polymer chains interact with solvent molecules and redox couple ions, effectively trapping them in hydrogel networks. This interaction leads to various effects such as modifying the ion‐solvation environment and enabling selective ion transport.

**Figure 2:**
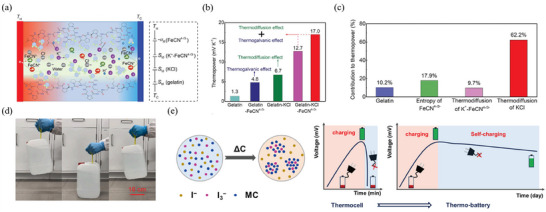
Recent research progress of THs based on gelatin and cellulose. a) Schematic diagram of the synergetic effect in Fe(CN)_6_^4−/3−^/KCl/gelatin. b) Comparison of the Seebeck coefficients of the pure gelatin, Fe(CN)_6_^4−/3−^/gelatin, KCl/gelatin, and Fe(CN)_6_^4−/3−^/KCl/gelatin. c) Fractional contribution to thermopower of Fe(CN)_6_^4−/3−^/KCl/gelatin. Reproduced with permission.^[^ ^38^ ^]^ Copyright 2020, The American Association for the Advancement of Science. d) Optical photograph of BC‐based hydrogel repeatedly lifts a water container weight up to 5000 g. Reproduced with permission.^[^ ^75^ ^]^ Copyright 2023, Elsevier. e) Schematic diagram of mechanism and self‐discharge behavior of the TEC based on I^−^/I_3_^−^/MC/BC. Reproduced with permission.^[^ ^25^ ^]^ Copyright 2024, The Royal Society of Chemistry.

Gelatin, a nature polymer, contains numerous function groups (e.g., ─COOH, ─NH_2_, and ─OH) that provide strong polarizability when subject to an electric field.^[^ ^70^, ^71^ ^]^ The negatively charged gelatin network facilities interactions between cations and the polymer chain, which creates a selective ion transport pathway. Leveraging this characteristic, Liu et al.^[^ ^38^ ^]^ designed a gelatin‐based device that achieved impressive thermoelectric conversion effects by combining redox couple with alkali metal salt (**Figure** **2**a). In this electrolyte, the Fe(CN)^4−/3−^ redox couple works based on the TG effect, while K^+^ and Cl^−^ function through TD effect. The Fe(CN)_6_^4−/3−^/gelatin system can exhibit a *S_i_* value of 4.8 mV K^−1^ based solely on TG effect. With the addition of KCl, the synergetic effect allows the Fe(CN)_6_^4−/3−^/KCl/gelatin system to achieve an impressive *S_i_* value of 17 mV K^−1^ after components regulation (Figure 2b). Notably, more than 60% of Seebeck coefficient in this system arises from TD effect of KCl (Figure 2c).

Cellulose, a widely abundant natural biopolymer on earth, features numerous hydroxy (─OH) groups with negative charges along its polymer chains, which makes it a suitable candidate for use in THs. Pringle et al.^[^ ^72^ ^]^ have developed the first cellulose‐based Fe(CN)_6_^4−/3−^ hydrogel electrolyte that contains 5% by weight of cellulose. This formulation achieves good mechanical properties with thermoelectrochemical performance comparable to that of a LE system (−1.38 mV K^−1^). Bacterial cellulose (BC), a member of the cellulose family, not only retains the intrinsic characteristics of cellulose, but also exhibits mechanical strength that is often lack in natural polymer, thereby making it a focus of recent research. Furthermore, due to its narrower fiber diameter (below 100 nm) and higher crystallinity (≈70–90%), BC can form a precise and robust network structure capable of accommodating an abundance of additional components.^[^ ^73^ ^]^ Additionally, the presence of nanochannels inside nanofibers allows pure natural BC hydrogel fibers to exhibit superior ionic conductivity compared to other hydrogels, such as PVA, PAAm, and gelatin.^[^ ^74^ ^]^ These characteristics make BC a promising matrix for THs.

In this context, Feng et al.^[^ ^75^ ^]^ prepared a TH by using BC confined with Fe(CN)_6_^4−/3−^ redox couple and hygroscopic salts. The resulting Fe(CN)_6_^4−/3−^/BC hydrogel not only facilitates heat‐to‐electricity conversion but also exhibits excellent mechanical properties. It is capable of repeatedly lifting a water container weight up to 5000 g without loosening or cracking (Figure 2d). In another study, Wang et al.^[^ ^25^ ^]^ used BC as the polymer matrix in hydrogel and harnessed the hydrophobic interaction between the methylcellulose and I_3_^−^ ions to achieve the n‐p conversion of I^−^/I_3_^−^ redox couple, obtaining thermopower values of 1.75 and −6.84 mV K^−1^, respectively. Notably, when the temperature gradient between the cold and hot side is removed, the device based on this TH can demonstrate self‐discharge behavior for up to 44 hours, which is attributed to the slow dissolution of MC/I_3_^−^ complexes (Figure 2e).

In addition to natural polymers, synthesized polymers also show potential for use in THs. PVA, as a synthesized polymer, features a simple molecular chain with a hydrophobic (CH_2_─CH_2_) backbone and hydrophilic (─OH) side groups. As early as 1987, Polak et al.^[^ ^76^ ^]^ made notable advancement by developing a polymer electrolyte (PVA/H_3_PO_4_) that exhibited good ionic conductivity. Moreover, PVA possesses unique characteristics, such as non‐toxicity, biocompatibility, and good water solubility, making it an ideal matrix for THs.

**Figure 3:**
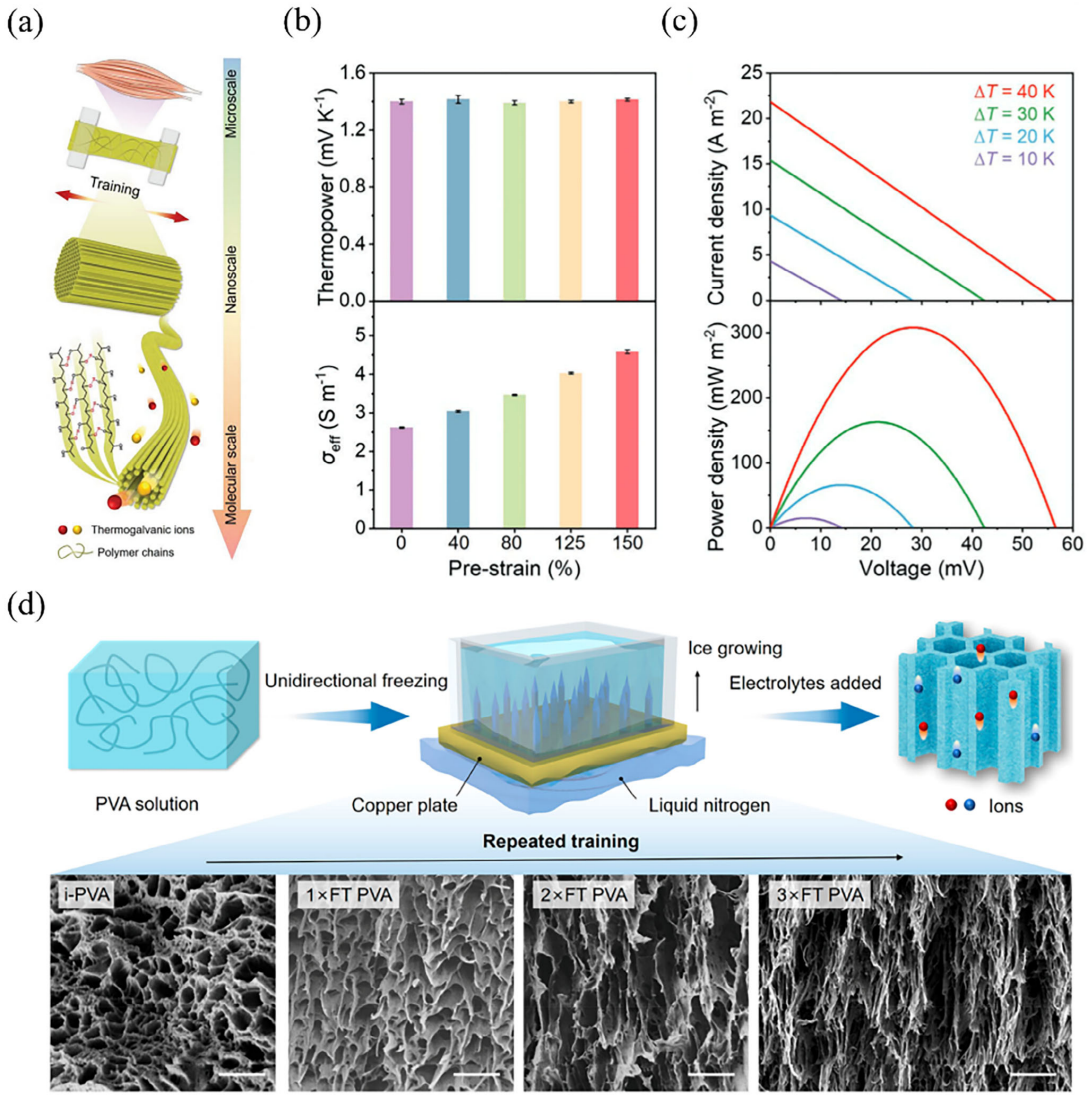
Recent research progress of THs based on PVA. a) Schematic illustration of bio‐inspired design and hierarchical structure of PVA hydrogel network. b) Thermopower and ionic conductivity of the PVA‐based TH after mechanical training at different pre‐strains. c) Current−voltage curve and output power density of the PVA‐based TH under different ∆T. Reproduced with permission.^[^ ^78^ ^]^ Copyright 2022, Wiley‐VCH. d) Schematic diagram and SEM images of the unidirectional freezing strategy to construct anisotropic PVA networks in TH. Reproduced with permission.^[^ ^79^ ^]^ Copyright 2022, American Chemical Society.

The earliest report on PVA‐based THs dates back to Zhou et al. in 2016,^[^ ^22^ ^]^ where the TH composite of PVA/Fe(CN)_6_^4−/3−^ and PVA/Fe^2+/3+^ demonstrates the *S_i_* of 1.21 and −1.02 mV K^−1^, respectively. Chemically cross‐linked PVA has been widely used as a matrix in hydrogels; however, its random and unorganized network structure limits ion transport, thereby reducing thermoelectrochemical power.^[^ ^77^ ^]^ Inspired by the natural muscles, Wu et al.^[^ ^78^ ^]^ used PVA as matrix to design an innovative TH (**Figure** **3**a). Through carefully designed mechanical training, the PVA/Fe(CN)_6_^4−/3−^ networks could self‐reorganize into a muscle‐like hierarchical structure, which significantly improves long‐term mechanical properties while maintaining the original thermopower. Remarkably, as the pre‐strain increases from 0 to 150%, the value of *S*_i_ remains relatively stable across all cases, and the ionic conductivity of TH significantly increases from ≈2.6 to 4.6 S m^−1^ (at room temperature). This improvement is attributed to aligned nanochannels that function as high‐ways for ion transport through PVA chains (Figure 3b). Consequently, the value of *P*_max_/(Δ*T*)^2^ reaches 0.15 mW m^−2^ K^−2^ (Δ*T* = 10 K) and 0.2 mW m^−2^ K^−2^ (Δ*T* = 40 K), significantly higher than other THs based on randomly cross‐linked PVA chains (Figure 3c). Similarly, Chen et al.^[^ ^79^ ^]^ employed the unidirectional freezing method to alter the original hydrogel network structure and enhance the thermoelectrochemical performance of PVA‐based TH. After three times freezing training (3 × FT), the structure of hydrogel transforms from random and isotropic to highly anisotropic, which facilitates molecular ions transport (Figure 3d). Consequently, the thermopower of PVA/Fe(CN)_6_^4−/3−^ slightly increases from ≈1.4 to ≈1.5 mV K^−1^, as a result of increased entropy difference of Fe(CN)_6_^4−/3−^ within the aligned nanochannels.

PAAm is another synthesized polymer matrix for THs. There are a large number of amide groups on the molecular chain that easily form hydrogen bonds with solvents like water.^[^ ^80^ ^]^ This ability enables PAAm to form hydrogel with high water content, which demonstrates good biocompatibility, resilience, elasticity, and biodegradability. Specific ions can strongly interact with PAAm. He et al.^[^ ^33^ ^]^ discovered that the addition of Fe(CN)_6_^4−/3−^ redox couple does not significantly affect the mechanical properties of PAAm. However, incorporating the Fe^3+^/Fe^2+^ redox couple increases its strain and stress by ≈1.8 times and ≈5 times, respectively, compared to pure PAAm. This phenomenon arises from the formation of a strong ionic cross‐linked network between high‐valent metal cations and amide groups along the PAAm chain.

**Figure 4:**
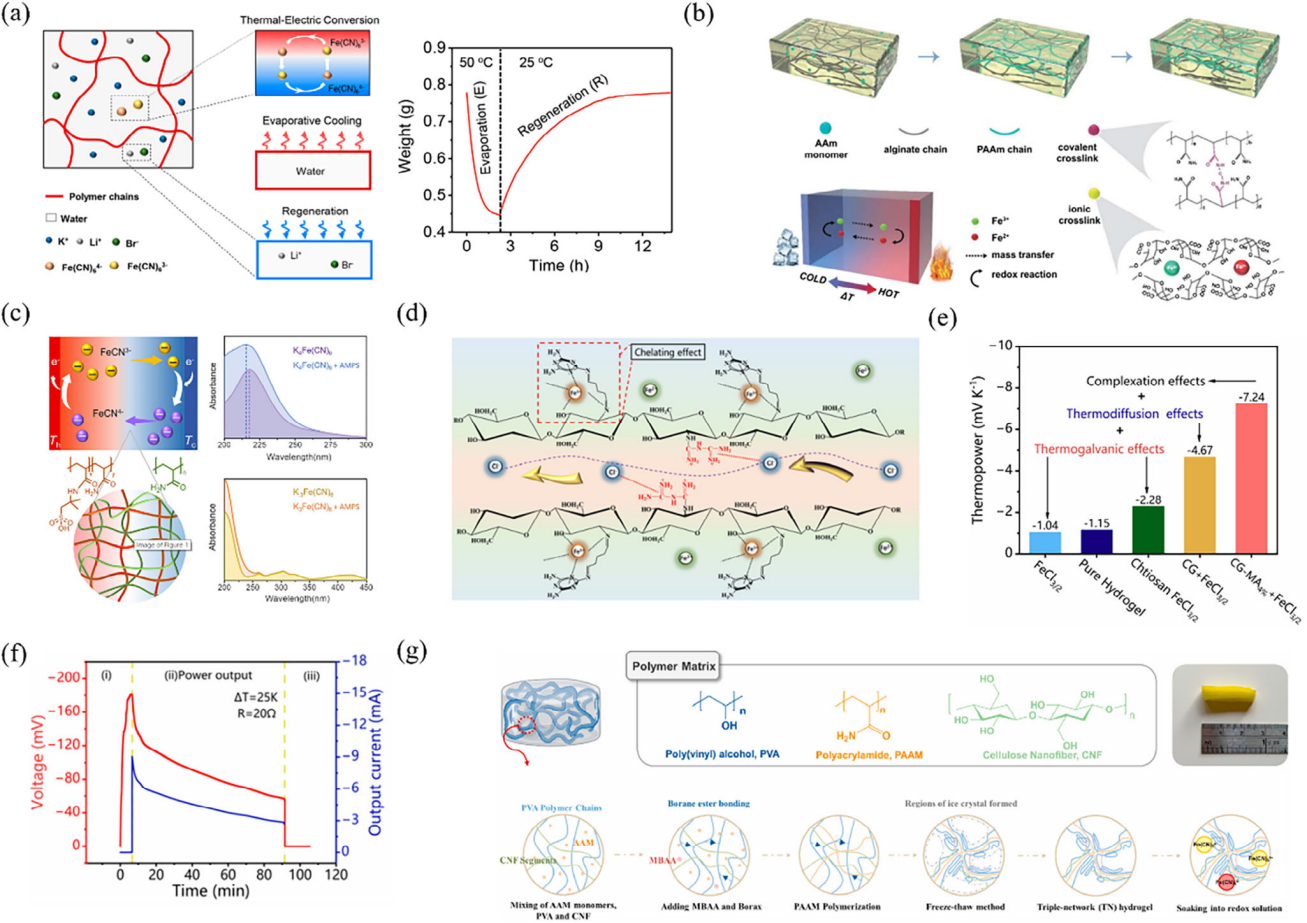
Recent research progress of THs based on PAAm. a) Schematic illustration of the composition and working principle of the smart hydrogel. Reproduced with permission.^[^ ^61^ ^]^ Copyright 2020, American Chemical Society. b) Cross‐linking structures and working mode in PAAM/SA/Fe^3+^/Fe^2+^. Reproduced with permission.^[^ ^27^ ^]^ Copyright 2022, Springer. c) Schematic illustration of composite polymer network and UV–vis spectra of the K_3_Fe(CN)_6_/K_4_Fe(CN)_6_ aqueous solution (with or without AMPS). Reproduced with permission.^[^ ^59^ ^]^ Copyright 2021, Elsevier. d) Schematic of the working mechanisms of dicyandiamide and MA. e) The value of thermopower of different ionic gel components. f) Recorded voltage and current curves of device's three operating stages with an external resistance of 20 Ω. Reproduced with permission.^[^ ^87^ ^]^ Copyright 2024, The Royal Society of Chemistry. g) Schematic of triple‐network TH preparation process and corresponding optical photo. Reproduced with permission.^[^ ^88^ ^]^ Copyright 2024, Elsevier.

Modifying the composition of PAAm‐based hydrogels can influence their properties, upon which unique functions are enabled. Chen et al.^[^ ^61^ ^]^ developed a smart PAAm‐based TH for efficient evaporative cooling and low‐grade heat harvest. This TH utilizes Fe(CN)_6_^4−/3−^ redox couple for heat‐to‐electricity conversion and hygroscopic LiBr for adaptive evaporation and absorption cycles. It demonstrates a viable method for stable harvest of waste low‐grade heat (**Figure** **4**a). When blended with ionically crosslinked sodium alginate (SA), the resulting double network SA/PAAm‐based hydrogels exhibited remarkable mechanical capabilities. Because of this, the composite hydrogel has been intensively investigated in TCCs.^[^ ^81^, ^82^ ^]^ Similarly, the SA/PAAm composite hydrogel is also an ideal matrix for THs. Liu et al.^[^ ^27^ ^]^ successfully developed a SA/PAAm‐based quasi solid‐state TEC, where Fe^3+^/Fe^2+^ redox couple in hydrogel not only resulted in the TG effect but also served as ionic crosslinkers (Figure 4b). This ionic cross‐linking network enhances ion transport through solvation structures, resulting in a *S*_i_ up to 1.43 mV K^−1^, and can significantly improve the mechanical properties of the TH.

Other polymers, such as PVDF and PVDF‐HFP, can serve as host for hydrogels due to their polar C−F chemical bonds and excellent liquid absorption capability.^[^ ^83^, ^84^ ^]^ Pringle et al.^[^ ^28^ ^]^ used PVDF and PVDF‐HFP as polymer matrix and incorporated water and the Co(bpy)_3_^2+/3+^ redox couple to prepare TH. They reported a high thermopower of ≈1.80–1.84 mV K^−1^.

In addition to the single polymer matrices, research on THs constructed by cross‐ linking polymer‐polymer combinations to enhance thermoelectrochemical performance has also gained attention. Wu et al.^[^ ^59^ ^]^ designed an TH by crosslinking 2‐acrylamide‐2‐methylpropane sulfonic acid (AMPS) and acrylamide (AM) monomers to create a hydrogel network. The composite hydrogel network favored interactions with redox couples, which results in an increased entropy difference and enhanced thermopower (Figure 4c). Similarly, Chen et al.^[^ ^85^ ^]^ selected a chaotropic comonomer of methyl chloride quaternized N, N‐dimethylamino ethylacrylate (DMAEA‐Q) and combined it with AM to constructed polymer‐network for TH. The quaternized cation has strong interactions with Fe^3+^ ion, which enlarges the entropy difference between Fe^3+/2+^ redox couple, thereby improving the thermopower based on the TG effect.

The contribution of TD effect to the thermopower generated by redox couple is relatively small and usually neglected.^[^ ^86^ ^]^ Recently, Li et al.^[^ ^87^ ^]^ proposed a novel strategy to increase thermopower and output power of TH by leveraging the synergy between TG and TD effect based on a single Fe^3+/2+^ redox couple. As shown in Figure 4d, the TH consists of chitosan/guanidine/melamine (CG‐MA)‐FeCl_3/2_ system featuring a double network structure. The positively charged N^+^ groups of guanidine increase the overall number of positive charges in the polymer backbone, which accelerates migration rate of Cl^−^ ions. The CG‐MA polymer network can be complexed with Fe^3+^ ions on the cold side due to the chelating effect of MA. This leads to an enhancement in both the entropy difference and concentration difference of Fe^3+^/Fe^2+^ redox couple. The thermopower attributed to the TG effect of the optimal CG‐MA_4%_‐FeCl_3/2_ increased from original value of −1.04 to −3.69 mV K^−1^, culminating a total value of −7.24 mV K^−1^ (Figure 4e). Notably, the TG effect in this TH is the primary contributor that accounts for over 50% of the total *S*_i_ value. Additionally, the 90‐min output energy density of the quasi‐solid‐state device reaches a record‐breaking 17.93 kJ m^−2^ (Δ*T* = 25 K) during continuous operation (Figure 4f).

The physical structure of the hydrogel network plays a crucial role in the performance of THs. Liu et al.^[^ ^88^ ^]^ constructed a triple‐network hydrogel composed of PAAm, PVA, cellulose nanofiber (CNF), and Fe(CN)_6_^4−/3−^ redox couple for the application in TECs. In this system, the PAAm network provides shape construction ability, the PVA network imparts flexibility, and the CNF network enhances the mechanical properties by penetrating into the other polymer networks (Figure 4g). After optimizing the component ratios, the resulting TH with an ideal hydrogel structure exhibits a high ionic conductivity 168 mS cm^−1^, a high PF of 47.9 µW m^−1^ K^−2^, and a ZT value of 0.06. This performance enhancement can be attributed to precise control of the nanostructures within the hydrogel matrix that can significantly improve ion transport.

### 4.2 Influence of Liquid Phase

In addition to the polymer matrix, the liquid phase, as a key component, significantly impacts THs. In the liquid phase, ions in solvent are complexed by solvent molecules, forming a solvation shell through charge–dipole interaction.^[^ ^89^ ^]^ This process alters the properties of redox couple ions and influences the thermochemical performance of the THs. Specifically, solvation impacts THs through two ways: 1) it changes the solubility of redox couple ions, which induces thermosensitive crystallization/precipitation of specific redox couple ions (e.g., Fe(CN)_6_^4−^). 2) It affects the entropy difference of redox couple ions by altering/reconstructing the solvation shell of redox ions.

**Figure 5:**
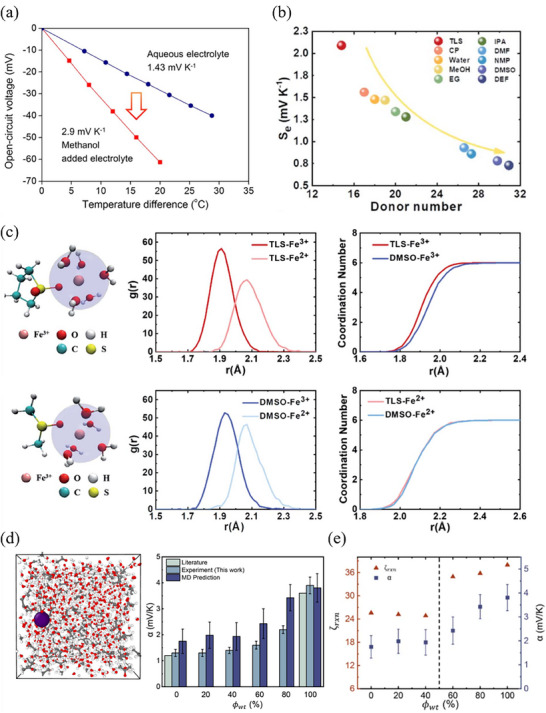
Influence of solvation structure on the thermopower of TECs. a) Significant elevated thermopower from the boosted structural entropy difference of Fe(CN)_6_^4−/3−^ redox couple reported by Kim et al. Reproduced with permission.^[^ ^43^ ^]^ Copyright 2024, Elsevier. b) *S*_i_ values of THs with various organic solvents with different DN. c) First solvation shell of the Fe^3+^ with TLS and DMSO was introduce into electrolyte, and the radial distribution function calculation results. Reproduced with permission.^[^ ^94^ ^]^ Copyright 2022, The Royal Society of Chemistry. d) Molecular dynamics prediction of thermopower in various electrolytes. e) Correlation between solvation order change and the temperature coefficients. Reproduced with permission.^[^ ^95^ ^]^ Copyright 2023, Wiley‐VCH.

In the case of liquid electrolyte, Kim et al.^[^ ^43^ ^]^ introduced organic solvents into a Fe(CN)_6_^4−/3−^ aqueous electrolyte to reorganize the solvation shell of redox couple. They demonstrated that adding solvents with Hildebrand solubility parameter ranging from 19.7 to 34.9 MPa^1/2^ could enhance the thermopower of electrolyte. Notably, the addition of methanol to the Fe(CN)_6_^4−/3−^ aqueous electrolyte significantly improved the thermopower from −1.43 to −2.9 mV K^−1^. This is attributed to reorganization of the solvation shell around Fe(CN)_6_^4−^, which increased the entropy difference between redox ions (**Figure** **5**a). Moritomo et al.^[^ ^90^ ^]^ systematically studied the impact of organic solvent on the Fe(CN)_6_^4−/3−^ aqueous electrolyte and proposed an empirical volume effect, highlighting the considerable influence of solvent on thermopower. In addition, Moritomo et al.^[^ ^91^ ^]^ explored solvent impact on the thermopower of FeCl_3_/FeCl_2_ and found that Fe^3+/2+^/acetone can achieve a thermopower of 3.6 mV K^−1^, nearly three times that of the pristine Fe^3+/2+^/water.

Fe^3+/2+^, as a redox couple without ligands, interacts more directly with solvent molecules. According to Lewis's acid–base theory, Fe^3+/2+^ ions acts as electron acceptor. The ability of solvent molecules to provide electron pairs can be quantified by the donor number (DN).^[^ ^92^, ^93^ ^]^ Solvents with varying DNs can modify the solvation structure compared to an purely aqueous environment, leading to changes in entropy difference between the redox couple ions. Sun et al.^[^ ^94^ ^]^ illustrated the impact of solvent molecules on solvation shell dynamics by examining solvents with different DN. They introduced organic solvents with different DNs into a Fe^3+/2+^/PAAm/water matrix to construct THs. The addition of tetramethylene sulfone (TLS), a solvent with high DN, reduced the *S*_i_ value to 0.74 mV K^−1^, while dimethyl sulfoxide (DMSO), a solvent with low DN, significantly increased the *S*_i_ value to 2.49 mV K^−1^ (Figure 5b).

The relationship between solvent DN and *S*_i_ can be interpreted from a theoretical perspective. In the TLS–based TH, the smaller radius of the Fe^3+^ ion solvent shell increases in entropy difference of Fe^3+/2+^ redox couple, leading to enhanced thermopower. Conversely, in the DMSO‐based TH, the larger radius of the Fe^3+^ ion reduces the entropy difference, resulting in lower thermopower (Figure 5c). The influence of solvation shell structure on the thermopower of redox couple can also be predicted using atomistic simulations. Yang et al.^[^ ^95^ ^]^ employed free energy perturbation (FEP) method in molecular dynamics (MD) simulations to quantify the partial entropy change. The predicted thermopower of the redox couple in liquid electrolyte agrees well with experimental results. For a water/acetone mixed system, they performed FEP computations and solvation structure analysis. The results confirm that the thermopower of Fe^3+/2+^ redox couple obtained experimentally aligns well with MD prediction (Figure 5d). Notably, only at high acetone fractions (*ϕ*_wt_ ≥ 60%), the thermopower significantly increases due to the significant change in the solvation shell order (Figure 5e).

**Figure 6:**
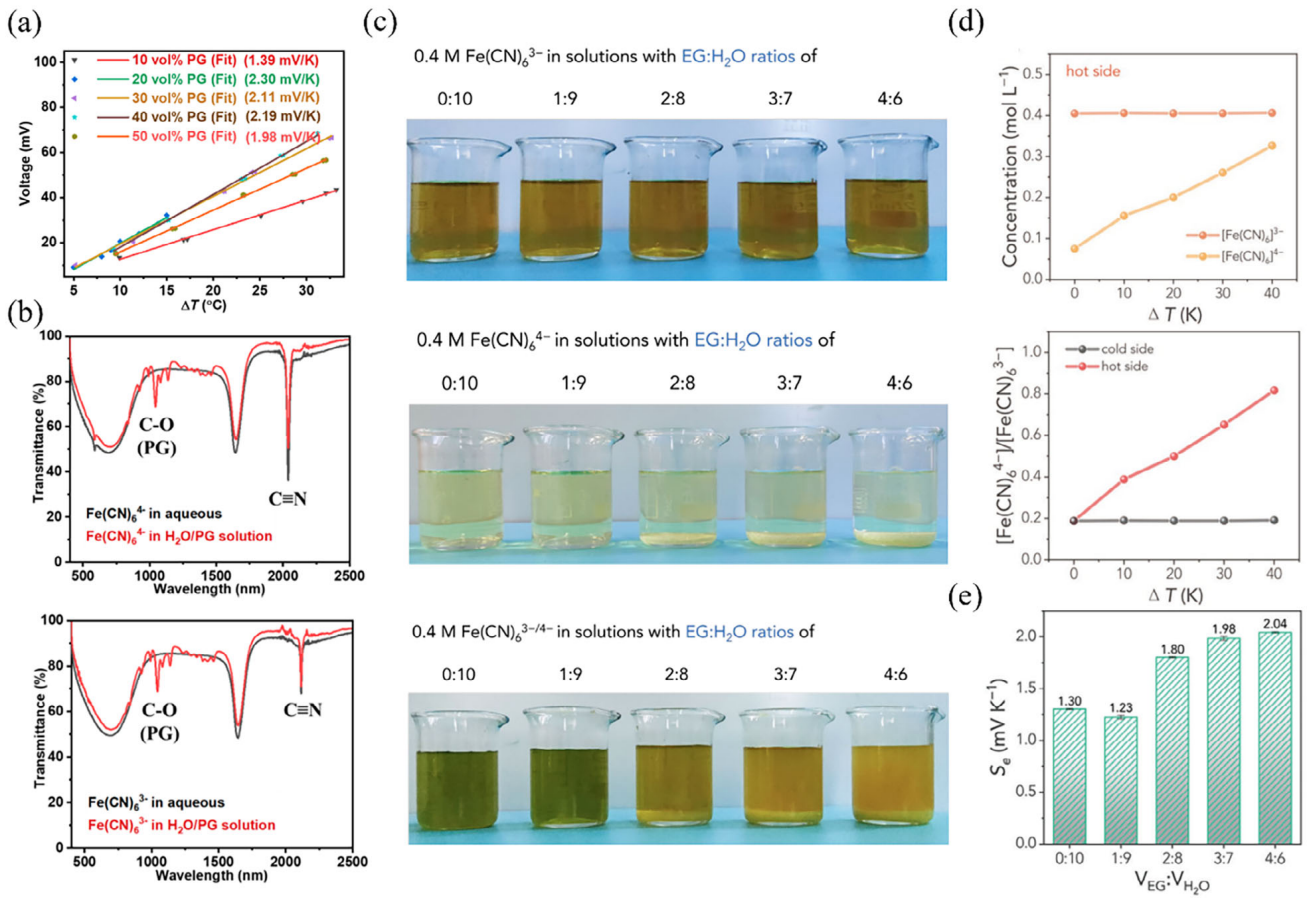
Influence of ion concentration on the thermopower of TECs. a) Seebeck coefficient of TECs with different PG content. b) FTIR spectra of 0.4 m Fe(CN)_6_^4−^ and Fe(CN)_6_^3−^ in aqueous and H_2_O/PG solutions. Reproduced with permission.^[^ ^73^ ^]^ Copyright 2023, Wiley‐VCH. c) Photos of 0.4 m Fe(CN)_6_^3−^, Fe(CN)_6_^4−^, and Fe(CN)_6_^4−/3−^ solutions with different EG:H_2_O ratios. d) The Fe(CN)_6_^4−/3−^ redox couple ions concentration and concentration ratio at hot and cold sides under different Δ*T*. e) Seebeck coefficient of the PAAm‐based hydrogel electrolyte with various EG:H_2_O ratios. Reproduced with permission.^[^ ^23^ ^]^ Copyright 2024, Wiley‐VCH.

When organic solvents are mixed with water in electrolytes, they not only lead to entropy difference by altering the solvation structure but also induce a concentration difference (ΔC_r_) in the redox couple ions, thereby enhancing the thermoelectrochemical performance of electrolytes. Wang et al.^[^ ^73^ ^]^ prepared BC hydrogels immersed in H_2_O/propylene glycol (PG) solution of Fe(CN)_6_^4−/3−^ for quasi solid‐state TEC. They found that PG triggered the gradual crystallization of K_4_Fe(CN)_6_ to create a ΔC_r_ between redox couple ions, which significantly increased the Seebeck coefficient from 1.39 to 2.30 mV K^−1^ (**Figure** **6**a). Furthermore, Fourier transform infrared (FTIR) spectra revealed that the C≡N characteristic peak of Fe(CN)_6_^4−/3−^ ions remained unchanged in the H_2_O/PG solution. This indicates that the Fe(CN)_6_^4−/3−^ ions in the mixed solution are preferentially solvated by water, with PG having no noticeable effect on their solvation (Figure 6b). These results suggest that ΔC_r_ is the primary factor for enhanced thermoelectrochemical performance of this system.

In a separate study, Liu et al.^[^ ^23^ ^]^ designed an ethylene glycol (EG)/PAAm‐based TH for quasi‐solid‐state TEC, which could operate continuously at sub‐zero temperature due to the EG's ability to prevent freezing by disrupting the hydrogen bonds between water molecules. Additionally, the solubility of Fe(CN)_6_^4−/3−^ redox couple ions in EG solvent varies significantly. As shown in Figure 6c, Fe(CN)_6_^3−^ ion did not precipitate in solutions with EG ratios ranging from 0 to 40 vol% at ambient temperature, while Fe(CN)_6_^4−^ ion began to precipitate at an EG ratio of 20 vol%. Moreover, the solubility of Fe(CN)_6_^4−^ increases with temperature, allowing it to dissolve at the hot side (Figure 6d). This creates a concentration gradient across the TH, which drives the migration of Fe(CN)6^4−/3−^ redox couple ions and introduces a crystallization/dissolution process that enlarges the entropy difference. Consequently, the PAAm‐based TH exhibits a remarkable increase in thermopower, from 1.30 to 2.04 mV K^−1^ (Figure 6e).

### 4.3 Use of Additives

Recent efforts in designing THs have significantly improved thermoelectrochemical performance in many systems by using various additives. In general, the entropy difference and concentration difference of a redox couple can be modulated by regulating the interactions between ions and additives. Guanidine salts, which rank highly in the chaotropic sequence, are known to destabilize non‐covalent bonding interactions. Among these, guanidinium chloride (GdmCl), the most widely used chaotrope, has been extensively investigated for its impact on water structures.^[^ ^96^ ^]^

**Figure 7:**
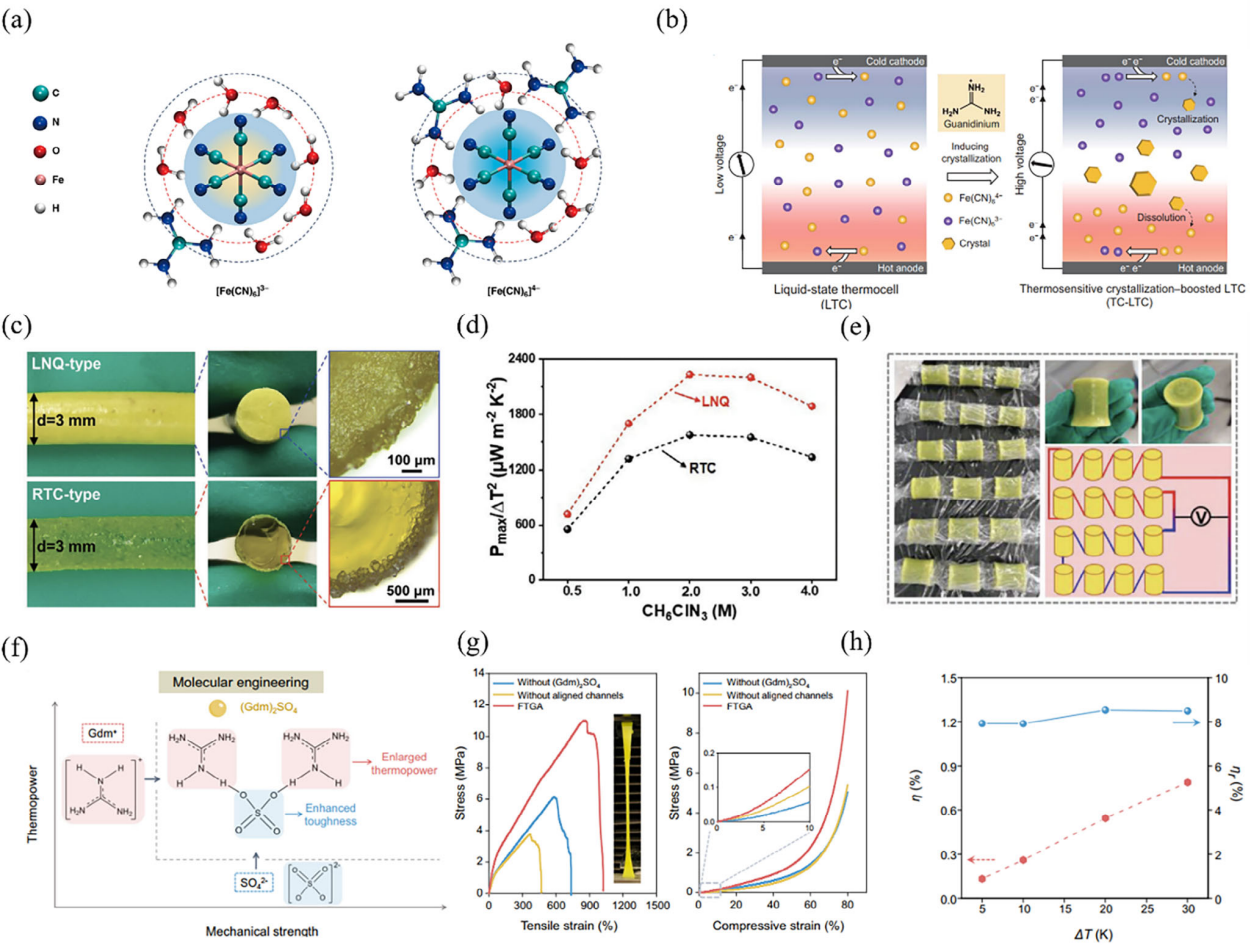
Effects of guanidine‐based additives. a) An illustration of the solvation forms of redox couple. Reproduced with permission.^[^ ^97^ ^]^ Copyright 2018, Springer. b) Schematic of thermosensitive dissolution/crystallization process. Reproduced with permission.^[^ ^98^ ^]^ Copyright 2020, The American Association for the Advancement of Science. c) The morphology comparison of different types of STHTs. d) The *P*_max_/Δ*T* ^2^ of the STHTs with increased of GdmCl concentrations. e) The digital photo of 16 hydrogel blocks connected devices. Reproduced with permission.^[^ ^100^ ^]^ Copyright 2024, Wiley‐VCH. f) Diagram of molecular engineering of (Gdm)_2_SO_4_ to influence the performance of FTGA. g) Stress–strain and compressive curves of different hydrogels. h) The Carnot‐relative efficiency of the FTGA (cold side was fixed at 298 K). Reproduced with permission.^[^ ^65^ ^]^ Copyright 2024, Springer.

Considering the Fe(CN)_6_^4−/3−^ redox couple as chaotropic anions, Zhou et al.^[^ ^97^ ^]^ reported that GdmCl acts as chaotropic cations, significantly interacting with the redox couple in aqueous electrolyte. Through chaotrope–chaotrope interactions, the solvation shells of Fe(CN)_6_^4−/3−^ redox couple undergo rearrangement, which enhances the structural entropy difference and nearly doubles the *S*_i_ value (**Figure** **7**a). In a subsequent study, a more intriguing property of GdmCl was uncovered.^[^ ^98^ ^]^ Utilizing the selective thermosensitivity of Gdm^+^ cation, they tuned the entropy difference and concentration difference of Fe(CN)_6_^4−/3−^ redox couple by the thermal crystallization/dissolution (Figure 7b). As a result, the device exhibits an outstanding Carnot efficiency (≈11.1%) and a high thermopower of 3.7 mV K^−1^. These discoveries highlighted Gdm salts as promising additives for THs due to their unique properties.

Building on Zhou's method for inducing crystallization, Ma et al.^[^ ^99^ ^]^ reported an impressive thermopower of 4.4 mV K^−1^ in the PAAm/SA/Fe(CN)_6_^4−/3−^ hydrogel by adding the GdmCl to promote Fe(CN)_6_^4−^ crystallization. Although the crystallized product can be confined and recycled within the hydrogel, the crystals tend to precipitate on the surface of hydrogel electrolyte, growing into large grains and resulting in low utilizing rates. To address this, Ma's group drew inspiration from the rapid cooling of metallic alloys and employed liquid nitrogen quenching (LNQ) strategy to enhance the performance of PAAm‐based TH.^[^ ^100^ ^]^ As shown in Figure 7c, hydrogel treated with LNQ exhibited significant differences in morphology compared to those with room temperature cooling (RTC). The LNQ‐type stretchable thermogalvanic hydrogel threads (STHTs) have only a few tiny crystals on their surface, attributed to the suppression of grain growth and the better dispersion of small grains in the hydrogel. Thanks to the efficient utilization of GdmCl, the maximum value of *P*_max_/Δ*T* ^2^ of LNQ‐type hydrogel reached 2227.5 µW m^−2^ K^−2^, a 41.5% increase over that of RTC‐type hydrogel (Figure 7d). Moreover, under a Δ*T* of ≈25 K, a device composed of 16 LNQ‐type hydrogel blocks achieved a relatively high voltage of 0.85 V, demonstrating its potential for practical application (Figure 7e).

Due to the diverse impact of GdmCl on the mechanical properties of hydrogel, researchers are investigating the application of other guanidine salts in the TH. In this context, Wang et al.^[^ ^65^ ^]^ proposed the use of a guanidine sulfate ((Gdm)_2_SO_4_) in TH to induce thermosensitive crystallization. As shown in Figure 7f, both the thermopower and the mechanical properties of hydrogel are enhanced. By combining this approach with directional freezing method to create aligned channels within PVA matrix, the resulting flexible thermogalvanic armor (FTGA) composed of PVA/Fe(CN)_6_^4−/3−^/(Gdm)_2_SO_4_ demonstrated both an outstanding Seebeck coefficient (5.58 mV K^−1^) and a high ionic conductivity (12.98 S m^−1^). Furthermore, thanks to its structure design and salting‐out effect of the SO_4_^2−^ anion, the FTGA exhibits excellent mechanical properties, which facilitates better integration with devices (Figure 7g). Importantly, the value of Carnot efficiency of FTGA, 8.53% near room temperature, surpassed the commercialization threshold (5%) (Figure 7h). Moreover, owing to high thermoelectrochemical performance and good mechanical properties, a large‐scale and flexible FTGA module made of 36 units was fabricated by 3D printing technology. When mounted on a human arm, the module generated a voltage of 0.58 V from body heat (Δ*T* ≈ 5 K).

**Figure 8:**
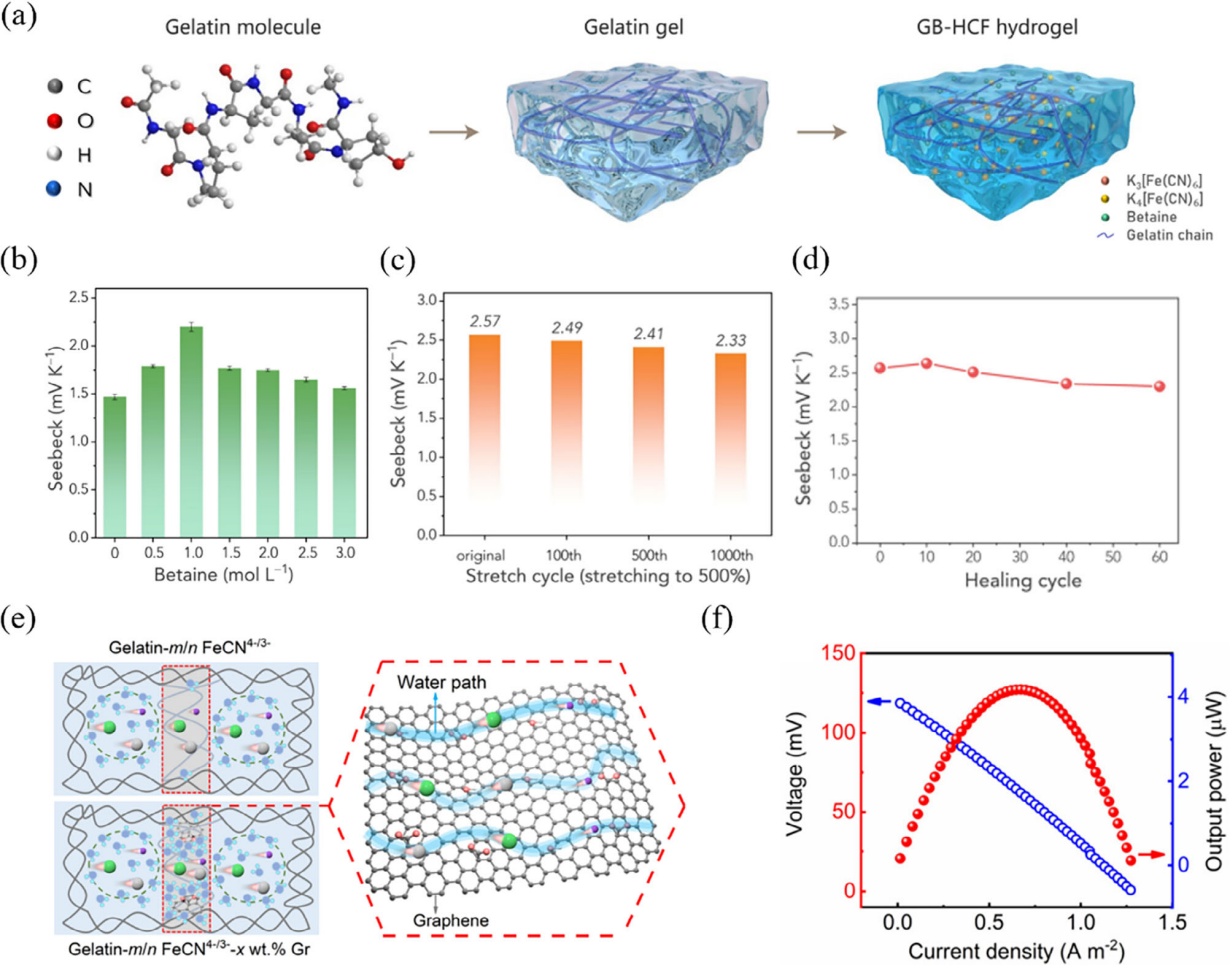
Effects of other additives. a) Diagram of the forming process of the gelatin/betaine/Fe(CN)_6_^4−/3−^ hydrogel. b) The influence of betaine concentration on the thermopower. Reproduced with permission.^[^ ^60^ ^]^ Copyright 2024, Wiley‐VCH. The thermopower of MXene‐boosted hydrogel after multiple c) stretching cycles at 500% strain and d) self‐healing cycles. Reproduced with permission.^[^ ^32^ ^]^ Copyright 2023, Springer. e) Schematic of ion diffusion in graph/gelatin/Fe(CN)_6_^4−/3−^ hydrogel. f) Power density‐voltage‐currently density curves for the device (four cells in series) at 323 K and Δ*T* = 3 K. Reproduced with permission.^[^ ^24^ ^]^ Copyright 2024, The Royal Society of Chemistry.

Recently, researchers have identified various other additives. Chen et al.^[^ ^60^ ^]^ employed straightforward one‐pot method to prepare a gelatin/betaine/Fe(CN)_6_^4−/3−^ TH (**Figure** **8**a). The abundant −N^+^(CH_3_)_3_ cation and −COO^−^ anion groups in betaine zwitterions can interact with the polymer chains to improve the mechanical properties of the TH and selectively rearrange the solvated structure of Fe(CN)_6_^3−^ to enlarge the entropy difference. As a result, the thermopower of gelatin‐based TH was boosted to 2.2 mV K^−1^ (Figure 8b). Additionally, MXenes, a new category of 2D transition metal carbides and nitrides materials, possess abundant surface terminations that impart unique functions.^[^ ^17^, ^101^ ^]^ Liu et al.^[^ ^32^ ^]^ combined the Ti_3_C_2_T*_x_* MXene nanosheet with polyacrylic acid (PAA)/GdmCl/Fe(CN)_6_^4−/3−^ hydrogel to create a new TH. It is found that numerous hydrogen bonds were formed between carboxyl groups of the PAA chain and polarized end groups of the MXene nanosheet, which effectively enhances the mechanical properties of hydrogel. Remarkably, even after 1000 cycles of 500% stretching, the composite TH maintained its integrity and exhibited high thermopower (Figure 8c). Moreover, MXene nanosheet endows TH with self‐healing capabilities. After 60 cut‐healing cycles, the thermopower of TH remained ≈2.30 mV K^−1^, corresponding to ≈90% of the initial value (Figure 8d). Consequently, the TEC based on the PAA/GdmCl/Fe(CN)_6_^4−/3−^/MXene hydrogel functions effectively as a self‐powered strain sensor. Stable thermopower with high repeatability and sensitivity over a wide strain range of 20% to 400% were produced.

Graphene, a widely used carbon material, can serve as not only an electrode but also an additive in THs. Niu et al.^[^ ^24^ ^]^ examined the effects of various forms of graphene, Gr, rGO, and GO, on the thermochemical performance in gelatin/Fe(CN)_6_^4−/3−^ hydrogel. Graphene acts as a “‘bridge”’ that connects surrounding free water clusters, facilitating quicker ion diffusion and thereby enhancing the thermopower associated with the thermodiffusion component (Figure 8e). As a proof‐of‐concept, the TH device, comprising four cells in series, produced an output power *P*_max_/(Δ*T*)^2^ = 1.2 mW m^−2^ K^−2^, which represents a record‐high value (Figure 8f).

### 4.4 Other Methods

Other methods for enhancing THs include fundamental composition design, specialized structure design, and the integration of photocatalysis, etc.

**Figure 9:**
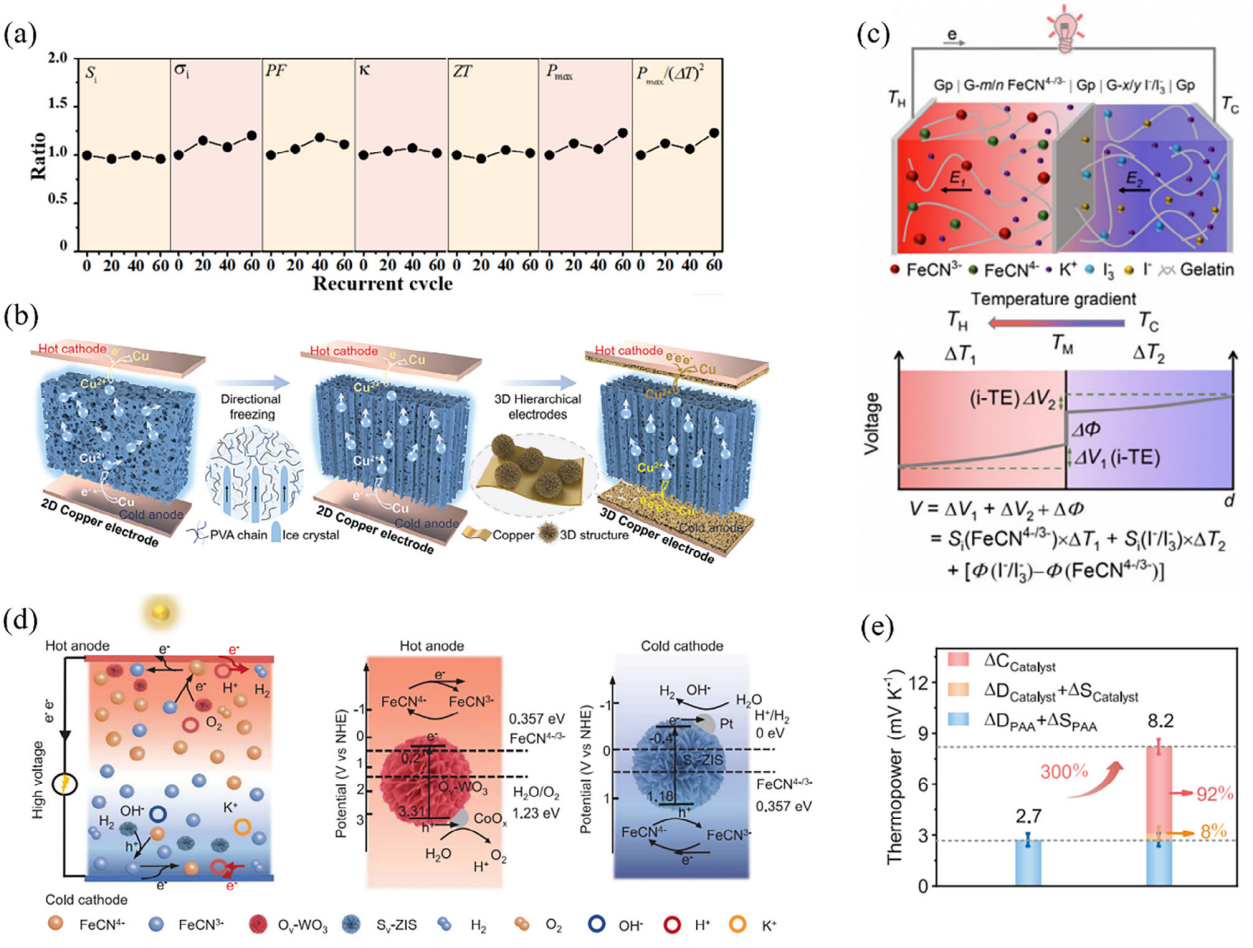
Other methods. a) The ratio of various parameters related to thermoelectrochemical performance for all‐inorganic TH after recurrent crush‐gelation to original. Reproduced with permission.^[^ ^102^ ^]^ Copyright 2024, The American Association for the Advancement of Science. b) Schematic of the synergetic effect of anisotropic PVA network and 3D hierarchical Cu electrode. Reproduced with permission.^[^ ^103^ ^]^ Copyright 2024, Wiley‐VCH. c) Schematic of double sandwich structure TEC under temperature gradient and the voltage distribution. Reproduced with permission.^[^ ^104^ ^]^ Copyright 2024, The Royal Society of Chemistry. d) Schematic diagram and working mechanism of in situ photocatalytically enhanced TEC. e) Thermopower of O_v_‐WO_3_/PPA/Fe(CN)_6_^4−/3−^/S_v_‐ZIS. Reproduced with permission.^[^ ^66^ ^]^ Copyright 2023, The American Association for the Advancement of Science.

In TECs, the THs have been utilized not only as aqueous or organic gels, but also as all‐inorganic gel. Huang et al.^[^ ^102^ ^]^ report the development of an all‐inorganic TH made by simply combining and stirring two inorganic salt solutions (FeCl_3_ and (NH_4_)_6_Mo_7_O_24_^.^4H_2_O). The reactions between various ions (e.g., Fe^2+^, NH^4+^, and MoO_4_^2−^) in mixed solution not only produce Fe^2+^ to form Fe^2+/3+^ redox couple for heat‐to‐electricity conversion but also facilitating the self‐assembly of hydrogel network through bond linkage. Moreover, after being completely crushed, this all‐inorganic hydrogel can re‐gel by reversibly destroying and repairing hydrogen bonds, which demonstrates highly stable thermoelectrochemical performances (**Figure** **9**a).

Collaborative construction of a hydrogel network and electrode structure design presents a promising approach to enhancing thermoelectrochemical performance of TECs. Gao et al.^[^ ^103^ ^]^ reported a high‐power n‐type TH that incorporates Cu^2+^/Cu^0^ redox couple into PVA hydrogel networks and leverages the synergistic engineering of Cu electrodes (Figure 9b). The fast ion transport channels in the anisotropic PVA hydrogel matrix, combined with high specific surface area 3D hierarchical Cu electrode, accelerated redox reaction kinetics and significantly increased output power by ≈15 times compared to the original TEC based on isotropic PVA and planar Cu.

By utilizing hydrogel structure design strategy, different redox couples can be integrated into a single TEC system to achieve high performance. In this context, Niu et al.^[^ ^104^ ^]^ designed a quasi‐solid‐state TEC featuring double sandwich structure of graphite paper (GP)|gelatin/Fe(CN)_6_^4−/3−^|GP|gelatin/I^−^/I_3_^−^|GP. The p‐type Fe(CN)_6_^4−/3−^ exhibits a negative electrode temperature coefficient, while the n‐type I^−^/I_3_^−^ has a opposite value. The electrode potential difference (ΔΦ) was incorporated into the TEC system through the association of asymmetric THs (Figure 9c). These asymmetric THs provided additional electrochemical energy to the TEC system. Consequently, more ionic thermoelectrochemical energy was generated and thermopower, output power density, and energy density were collaboratively improved.

In certain cases, catalysis can significantly enhance the performance of TECs as well. Li et al.^[^ ^66^ ^]^ proposed an in situ photocatalysis‐assisted integrated system (WO_3_ photocatalyst containing CoO_x_ (O_v_‐WO_3_)/polyacrylic acid‐based TEC/Pt‐containing ZnIn_2_S_4_ (S_v_‐ZIS)) to boost the redox reaction. As illustrated in Figure 9d, O_v_‐WO_3_ serves as an O_2_‐evolution photocatalyst (OEP) in the upper layer of PAA matrix, which facilitates the forward reaction from FeCN^3−^ to FeCN^4−^ and promoting the generation of O_2_ from H_2_O. This results in a high amount of FeCN^4−^ on the hot side. Conversely, the S_v_‐ZIS functions as a H_2_‐evolution photocatalyst (HEP) in the lower layer of the PAA matrix. On the cold side, FeCN^4−^ converts back to FeCN^3−^ and H_2_ is generated from H_2_O, wherein FeCN^3−^ concentration is increased. Under light irradiation situations, the formation of a continuous concentration gradient of the redox couple leads to a significant improvement in the thermopower of O_v_‐WO_3_/PPA/Fe(CN)_6_^4−/3−^/S_v_‐ZIS system, which increases 2.7 to 8.2 mV K^−1^, an enhancement of ≈3 folds (Figure 9e). The comprehensive data provided in **Table** **2** offers a detailed overview of the thermoelectrochemical performance of various THs.

**Table 2:** A summary of performance metrics of reported THs.

Materials	Redox couple	Ionic conductivity (*σ*_i_) [S m^−1^]	Thermopower *(S*_i_) [mV K^−1^]	*P*_max_/Δ*T* ^2^ [mw m^−2^ K^−2^]	Refs.
Gelatin/KCl	K_4/3_Fe(CN)_6_	/	17.0	/	[38]
Cellulose	K_4/3_Fe(CN)_6_	/	1.38	/	[72]
BC	K_4/3_Fe(CN)_6_	/	1.41	≈0.03	[75]
BC/MC	I^−^/I_3_^−^	/ /	1.75 −6.84	/	[25]
PVA	K_4/3_Fe(CN)_6_ FeCl_3/2_	≈1 ≈0.6	1.21 −1.02	/	[22]
PVA	K_4/3_Fe(CN)_6_	4.6	1.40	0.20	[78]
3FT‐PVA	K_4/3_Fe(CN)_6_	4.6	1.50	0.22	[79]
PAAm	K_4/3_Fe(CN)_6_ Fe(ClO_4_)_3/2_	≈1.3 ≈6.5	1.37 −1.65	0.31 0.4	[33]
PAAm/SA	FeCl_3/2_	≈0.4	1.43	/	[27]
PVDF	Co(bpy)_3_^3+/2+^	/	1.80	0.0144	[28]
PAAm/AMPS/NaCl	K_4/3_Fe(CN)_6_	12	1.60	0.61	[59]
PAAm/DMAEA‐Q	FeCl_3/2_	≈1	2.02	0.10	[85]
CG/MA	FeCl_3/2_	12.78	−7.24	/	[87]
PAAm/PVA/CNF	K_4/3_Fe(CN)_6_	16.8	1.69	/	[88]
BC/PG	K_4/3_Fe(CN)_6_	0.53	2.30	/	[73]
PAAm/EG/MXene	K_4/3_Fe(CN)_6_	1.65	2.04	0.160	[23]
PAAm/SA/GdmCl	K_4/3_Fe(CN)_6_	10.5	4.4	1.78	[99]
LNQ‐type STHTs	K_4/3_Fe(CN)_6_	8.8	4.5	2.27	[100]
FTGA	K_4/3_Fe(CN)_6_	12.98	5.58	11.9	[65]
Gelatin/Betaine	K_4/3_Fe(CN)_6_	3.5	2.2	0.48	[60]
PAA/MXene/GdmCl	K_4/3_Fe(CN)_6_	≈4.3	2.57	/	[32]
All‐inorganic TH	FeCl_3/2_	9.3	1.64	0.00273	[102]
Gelatin/Graphene	K_4/3_Fe(CN)_6_	0.20	13.0	1.2	[104]
PPA/O_v_‐WO_3_	K_4/3_Fe(CN)_6_	/	8.20	8.50	[66]

## 5 Emerging Application Aspects

Compared to traditional electron‐based thermoelectric materials that rely on the electronic Seebeck effect, TECs utilizing the thermogalvanic effect can also directly convert heat into electricity, while exhibiting significantly higher thermopower. TH possesses excellent mechanical properties, high thermopower output, and low temperature gradient requirements, which makes them well‐suited for fabricating quasi‐solid‐state TECs targeting low‐grade heat harvesting. Very often, a single module cannot generate adequate output voltage for practical applications due to a slight temperature gradient involved. To address this, multiple units are usually connected in series or parallel to generate a constructed ionic thermoelectric device. This enables the generation of useful thermovoltage (> 1.0 V) to meet diverse application requirements.^[^ ^105^ ^]^

**Figure 10:**
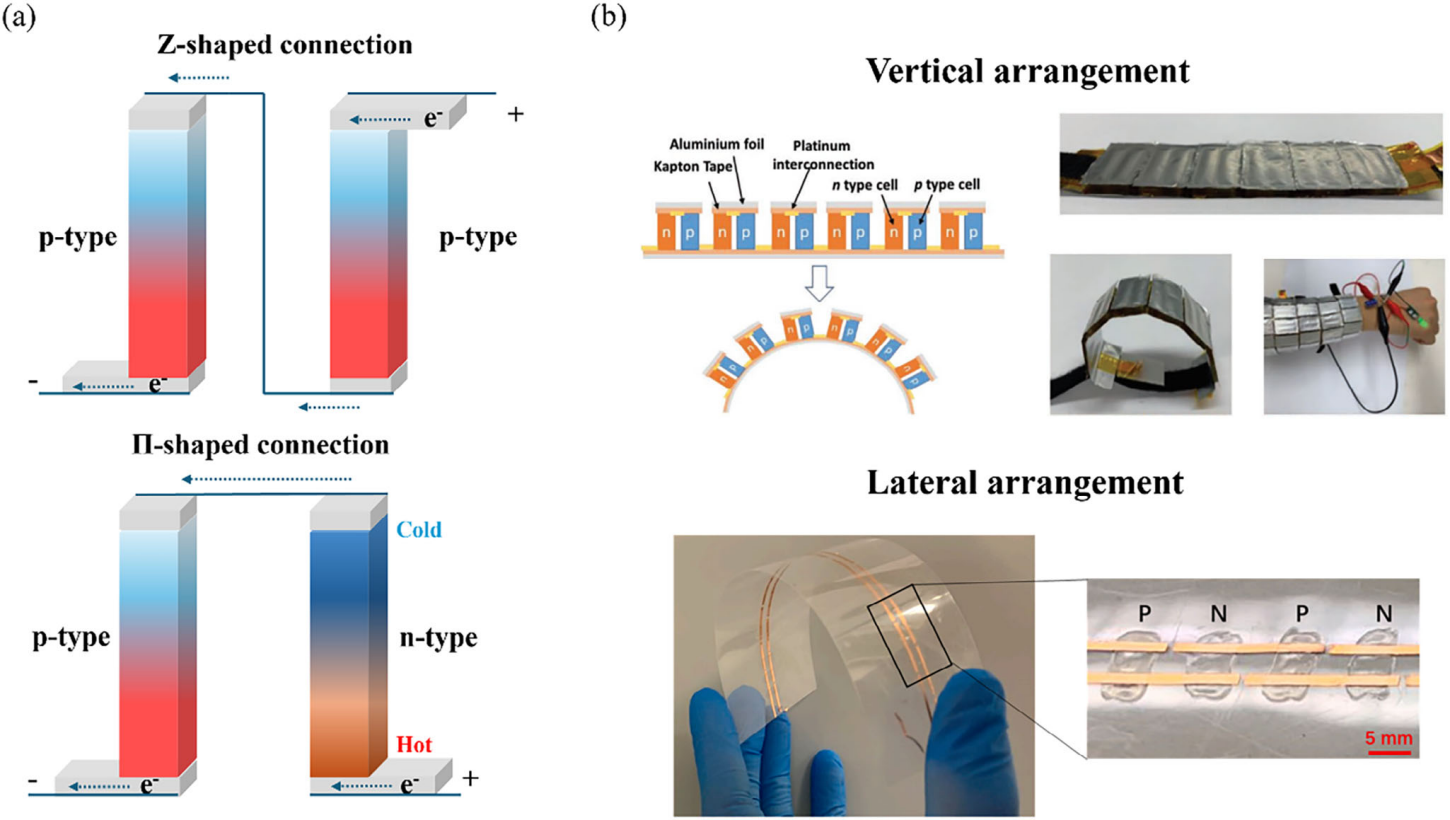
The TH‐based device integration mode. Schematic diagram of a) the connection methods of single module b) and ionic thermoelectric devices. Reproduced with permission.^[^ ^106^, ^107^ ^]^ Copyright 2022, Elsevier.; Copyright 2020, Wiley‐VCH.

Typical integrated TEC devices can be constructed by two primary methods to connect single modules to enable high power density and output voltage (**Figure** **10**a). The first method, known as Z‐shaped connection, is suitable for devices using either p‐ or n‐type materials. The second method, called Π‐shaped connection, is designed for combining the p‐ and n‐type materials. One of the most promising applications of ionic thermoelectrochemical devices is power generation, which harnesses energy from the small temperature gradient in our surrounding environment. These self‐powering ionic electricity devices can be categorized into vertical or lateral structures based on their design (Figure 10b). By integrating both n‐type and p‐type materials into a single module array, the internal circuit of the device is simplified.

### 5.1 Energy Harvesting

**Figure 11:**
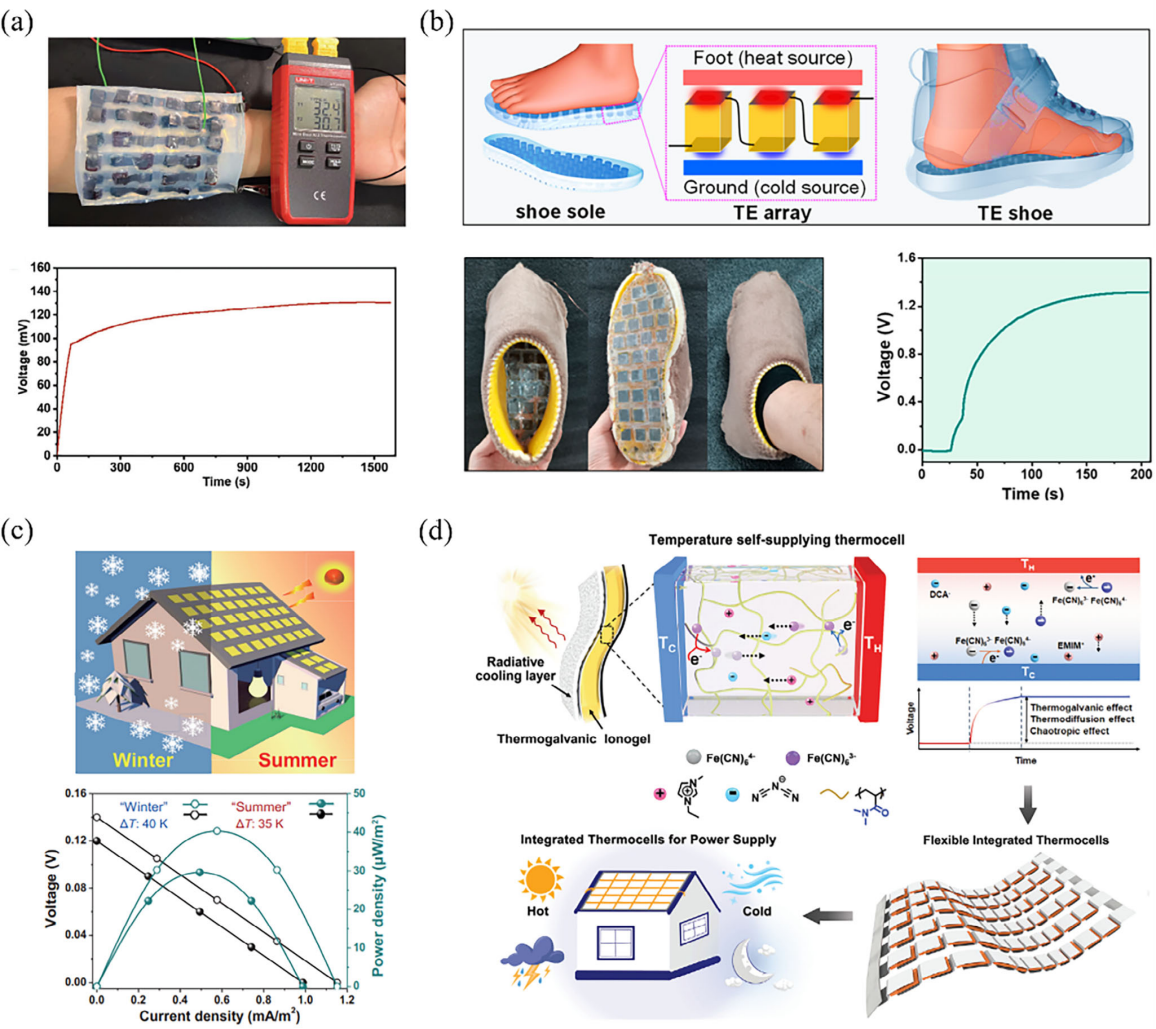
Performance of self‐power devices based on THs. a) Implementation of the device to the arm generates a continuous and stable voltage output. Reproduced with permission.^[^ ^109^ ^]^ Copyright 2024, Wiley‐VCH. b) Wearable thermoelectric shoes for waste heat harvesting. Reproduced with permission.^[^ ^110^ ^]^ Copyright 2023, American Chemical Society. c) Illustration of generated power of the smart house in simulative winter and summer. Reproduced with permission.^[^ ^111^ ^]^ Copyright 2024, Springer. d) A diagram of the thermogalvanic ionogel‐based TEC for all‐weather power production. Reproduced with permission.^[^ ^112^ ^]^ Copyright 2024, Wiley‐VCH.

Human body heat is a readily available natural heat source with enormous potential for directly powering wearable devices by leveraging the temperature gradient between the human body and its surroundings. In this context, Zhou et al.^[^ ^22^ ^]^ first demonstrated the feasibility of a self‐powering vertical device using an TH composed of 59 pairs of PVA/Fe(CN)_6_^4−/3−^ (p‐type) and PVA/Fe^3+/2+^ (n‐type) THs. They developed a proof‐of‐concept wearable TEC capable of generating an output voltage up to 1.0 V by using the human body as the energy source. Similar application have been explored in other studies.^[^ ^33^, ^108^ ^]^ Recently, Chen et al.^[^ ^109^ ^]^ demonstrated a wearable device based on TH that continuously and stably utilizes a small temperature gradient of 1.7 K between the human arm skin and the surrounding environment to generate power (**Figure** **11**a). In another study, Ma et al.^[^ ^110^ ^]^ developed a highly flexibility and antifreezing PAAm/SA/EG/Fe(CN)_6_^4−/3−^ TH, which was incorporated it into a wearable thermoelectric shoe for harvesting human body heat in a simulated cold environment of −30 °C (Figure 11b).

The temperature difference between human living environment and the natural environment also holds significant potential for energy utilization. During summer or winter, the external temperature of buildings becomes relatively high or low. This creates a substantial temperature gradient between indoor and outdoor spaces. In this context, Ma et al.^[^ ^111^ ^]^ proposed that the TH‐based devices have promising applications in smart buildings for energy supply, particularly in cities with extreme hot and cold climates (Figure 11c).

Another abundant natural heat source is solar radiation, which could be harnessed as an energy source for TECs, as sunlight produces a heat flux density of 1 kW m^−2^ on Earth's surface. Yan et al.^[^ ^112^ ^]^ developed an all‐weather, temperature self‐supplying ionic thermoelectric device that combines a thermogalvanic ionogel‐based TEC with passive radiative cooling to sustainably generate electricity sustainably (Figure 11d). The integrated TECs, comprising of 80 units, delivered a voltage of > 45 V under the solar intensity of 1000 W m^−2^ and achieved a higher *P*_max_/∆*T* ^2^ of 25.84 mW m^−2^ K^−2^.

### 5.2 Sensors

**Figure 12:**
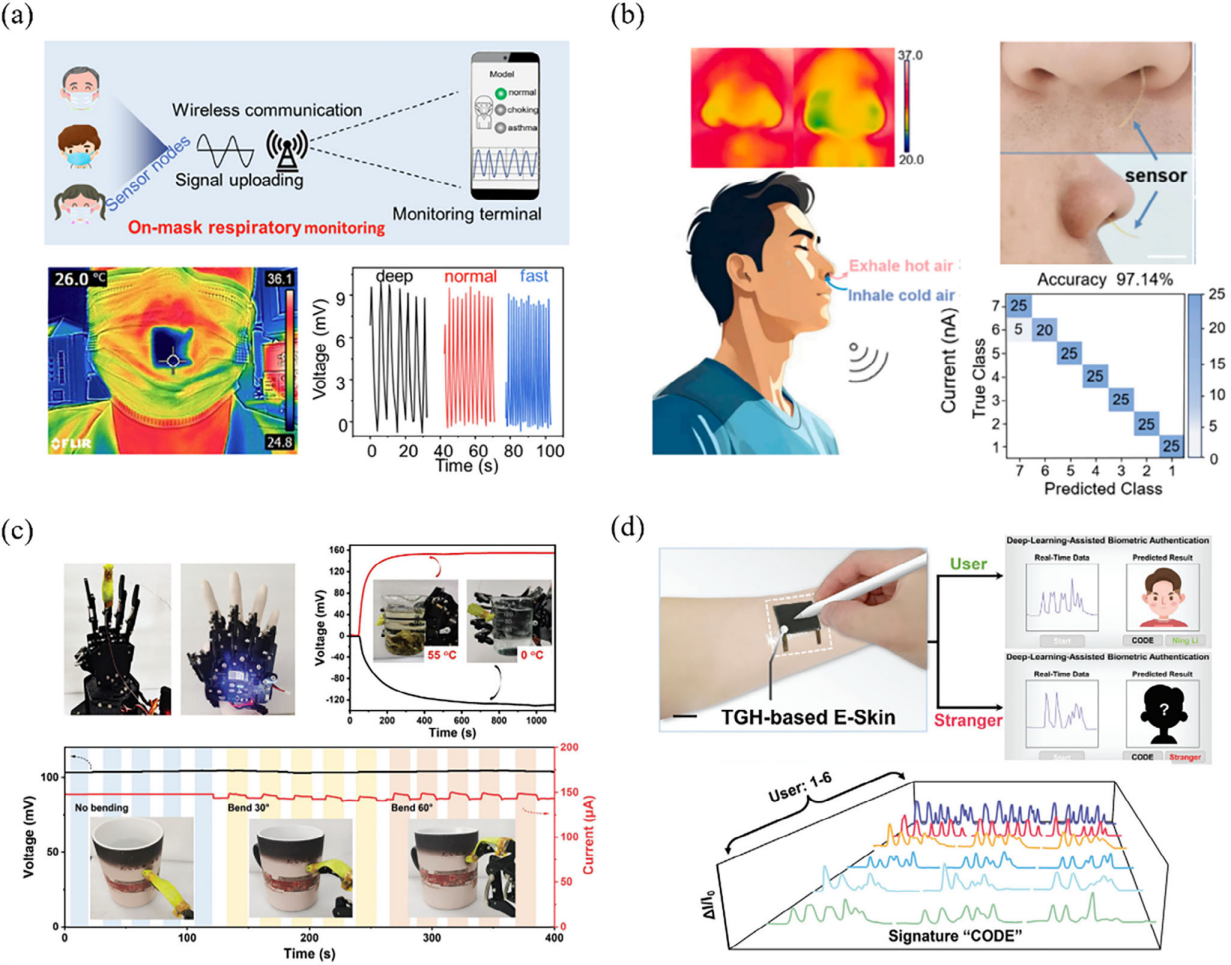
Performance of sensor devices based on THs. a) Demonstration of mask respiratory monitoring based on thermogalvanic hydrogel. Reproduced with permission.^[^ ^113^ ^]^ Copyright 2022, American Chemical Society. b) Schematic diagram of self‐powered nasal thermoelectric sensor assisted by machine learning for respiratory monitoring. Reproduced with permission.^[^ ^115^ ^]^ Copyright 2025, Elsevier. c) Flexible hydrogel patch sensors are attached to the fingers of a robot hand and output voltage curves in various situations. Reproduced with permission.^[^ ^116^ ^]^ Copyright 2023, Wiley‐VCH. d) Illustration of different persons after writing down the word “CODE” on the electronic skin. Reproduced with permission.^[^ ^117^ ^]^ Copyright 2024, Wiley‐VCH.

THs have also gained significant attention in the fields of sensors due to their sensitivity to temperature and heat flux. They can detect temperature variations, movements, and physiological activities in wearable devices, electronic skin, and other applications. For example, Zhang et al.^[^ ^113^ ^]^ developed TH patches integrated into mask by leveraging the temperature gradient between the surrounding and the heat produced by human breathing, as shown in **Figure** **12**a. These gel patches act as sensors that effectively convert physiological data (e.g., normal, deep, and fast breaths) into straightforward electrical pulse signals. In another work, Zhang's group fabricated a highly sensitive temperature‐monitor sensor using hydrogel with Fe^3+/2+^ as redox couple.^[^ ^114^ ^]^ The device produced distinct current signals in response to alterations in the user's body temperature, which provides critical information related to the wearer's physiological state. Furthermore, Guo et al.^[^ ^115^ ^]^ combined deep learning strategy with a self‐powered in‐nostril fiber sensor based on PVA/Fe^3+/2+^ TH (Figure 12b). When breathing occurs, the temperature difference in the nasal cavity is converted into electrical signals. By analyzing these signals with deep learning algorithms, seven typical breathing modes can be identified with an accuracy rate of up to 97.1%. These studies highlight the potential of THs for monitoring human activities and foreseeing potential health problems.

Moreover, THs can simultaneously respond to strain and temperature. For instance, Ma et al.^[^ ^116^ ^]^ developed a high‐strength stretchable PVA/Fe(CN)_6_^4−/3−^ hydrogel for temperature sensing in manipulator fingers. As shown in Figure 12c, when the TH attached to a finger, it generates distinct electrical signals upon contact with objects at different temperatures. The bending of the TH relaxes the cross‐linked polymer network, which reduces resistance and enables strain sensing. Similarly, Sun et al.^[^ ^26^ ^]^ created a PAAm/single‐walled carbon nanotube/Sn^4+/2+^ TH capable of monitoring human finger, wrist, and elbow movement with high sensitivity. In another study, Zhang et al.^[^ ^117^ ^]^ designed a self‐powered electronic skin with a sandwich structure, in which thermogalvanic hydrogel was used as the functional layer. Based on variations in writing intensity and stroke, the current signals collected from the e‐skin exhibit distinct peak patterns, which can be used for signature recognition and biometric authentication (Figure 12d).

### 5.3 Integration with Existing Energy Technologies

TH‐based TECs face integration challenges due to mismatched output characteristics (voltage/current) for conventional electronics and performance dependence on specific operating conditions (e.g., temperature gradients). Existing energy technologies can help to solve these challenges. In 2019, Zhou et al.^[^ ^118^ ^]^ developed a tandem device consisting of a polypyrrole (PPy) broadband absorber/radiator, a TEC, and thermal storage materials (Cu foam/polyethylene glycol 1000) that could harvest environmental thermal energy all day. The PPy layer efficiently heats during the day and cools down at night, while Cu foam/PEG1000's phase transitions enable the TEC to recycle thermal energy and continuously generate electricity. This hybrid system achieved 0.6 W m^−2^ power output under simulated sunlight and 53 mW m^−2^ at night, thus showing promise for continuous energy harvesting. In 2023, Li et al.^[^ ^66^ ^]^ designed a TEC‐photocatalysts hybrid system. By using separate photocatalysts on hot and cold sides, it enhances redox ions conversion under light, thus achieving a high output power *P*_max_/(Δ*T*)^2^ = 8.5 mW m^−2^ K^−2^ for solar thermal energy harvesting. Also, it enables simultaneous hydrogen and oxygen production via photocatalytic water splitting. In 2024, Zhang et al.^[^ ^119^ ^]^ developed an asymmetric lithium‐ion TEC with a Li‐metal anode in cold side and a graphene cathode in hot side. This innovative system not only enables energy storage but also offers a promising approach for harvesting low‐grade heat and converting it into electricity. In addition, combining TH‐based TECs with another complementary energy harvesting technologies like triboelectric nanogenerators (TENGs) could also overcome some of these limitations. TENGs effectively convert high‐entropy, low‐frequency mechanical energy into electricity,^[^ ^120^, ^121^, ^122^ ^]^ while TH‐based TECs thrive at thermal energy conversion. Their integration enables hybrid systems that simultaneously harvest multiple energy sources, hence improving the overall conversion efficiency and system reliability.

Furthermore, TENGs demonstrate exceptional performance as mechanical sensors, capable of detecting pressure, vibration, and motion via triboelectrification effects.^[^ ^121^, ^122^, ^123^ ^]^ Integrating TH‐based TECs with TENGs enables multiparameter sensors capable of simultaneous monitoring. For environmental applications, such hybrid devices could measure temperature, wind speed, and rainfall intensity by combining both energy conversion mechanisms. This integration approach could be extended to electronic skins (E‐skins),^[^ ^124^, ^125^, ^126^ ^]^ where combining TECs with piezoelectric nanogenerators enables multifunctional, self‐powered sensing systems. Overall, by leveraging complementary strengths, these hybrid technologies can overcome integration challenges while expanding application possibilities.

## 6 Conclusions and Outlook

**Figure 13:**
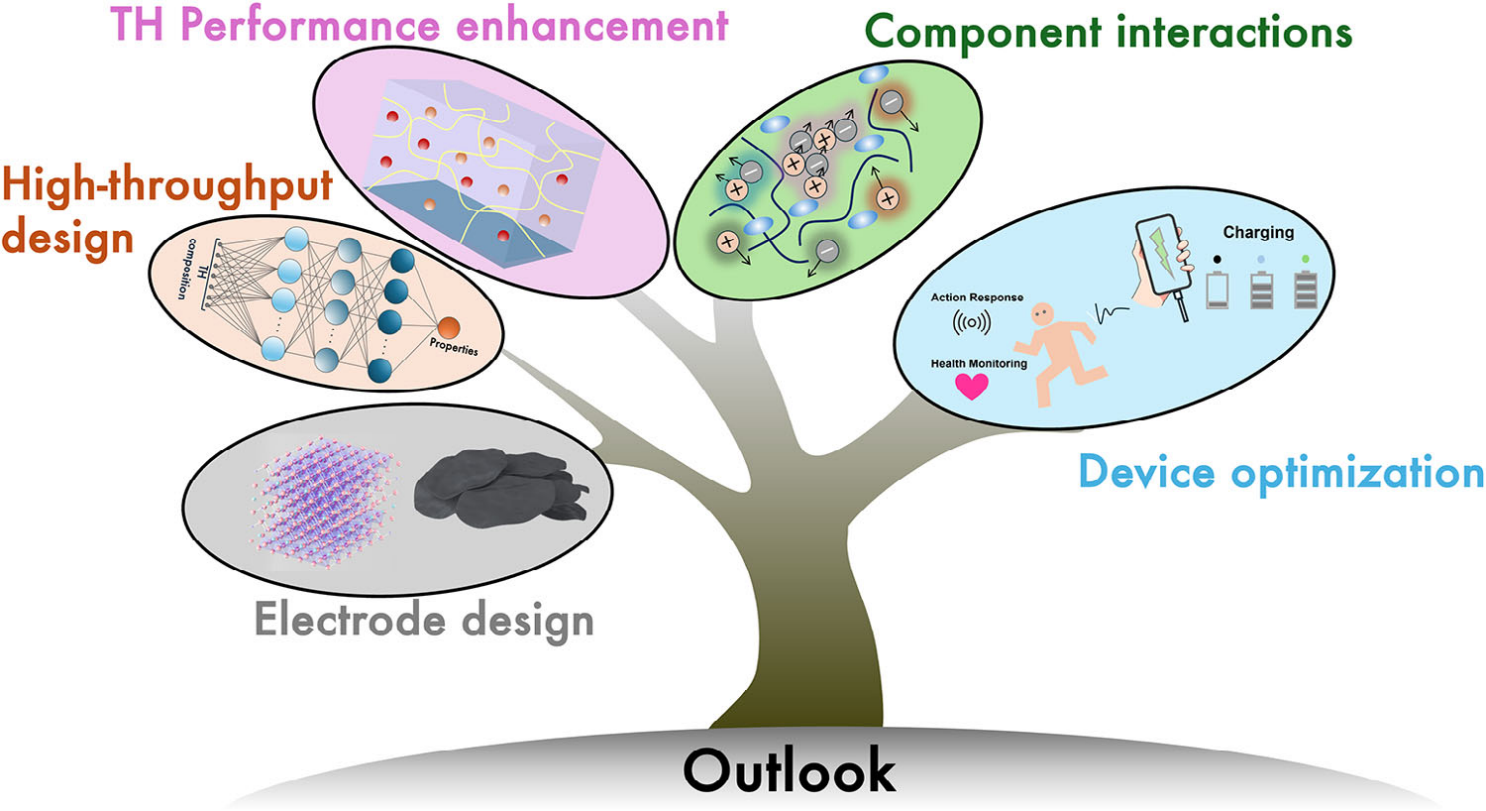
Key challenges, potential future directions and applications for THs.

In summary, THs constitute a transformative advancement in ionic thermoelectric material design that offers a sustainable pathway for green energy conversion. Unlike electronic thermoelectric materials, THs exhibit high thermopower, which makes them ideal solutions for harvesting the low‐grade waste heat. Their unique combination of mechanical flexibility, biological compatibility, and environmental sustainability enables diverse implementation scenarios across multiple technology domains. This review systematically summarizes the ionic thermoelectric conversion mechanisms and recent progress in TH research. Special emphasis is placed on strategies that enhance thermoelectrochemical performance, including polymer matrix, liquid phase composition, and functional additives. Additionally, key optimization strategies such as inorganic TH design, specialized structure design, and the integration of photocatalysis are discussed. Beyond material innovation, we also examine the diverse applications of THs spanning self‐power devices, sensors, and other emerging applications. Despite significant progress, research on THs is still in its early stages. The key challenges, potential future directions, and applications for THs are outlined in **Figure** **13**.

- Enhancing the performances of THs: High‐performance THs are still in development. Key areas for improvement include finding new n‐type/p‐type electrolyte systems, balancing thermoelectrochemical performance with mechanical properties, and enhancing hydrogel stability. To date, the Fe(CN)64−/3− redox couple is the most extensively studied due to its relatively high thermopower, but there is a pressing need to identify new redox couples with stronger TG effects to boost the thermoelectric performance. Furthermore, similar to electronic thermoelectric materials, simultaneously increasing the Seebeck coefficient and ionic conductivity of THs remains an unprecedented challenge. Further research into the interplay between the two parameters is essential to improve the efficiency of quasi‐solid‐state TECs. Additionally, since TECs must operate continuously, the stability of THs remains a key concern. Existing research has developed durable and robust THs by optimizing their mechanical properties, aiming to maintain stable operation under practical applications. For example, Liu et al.^[^ ^32^ ^]^ successfully constructed a robust and self‐healable TH by combining Ti3C2Tx MXene and PAA, which exhibits high thermopower even after 1000 stretching cycles and 60 cut‐healing cycles. Besides, Liu et al.^[^ ^23^ ^]^ and Chen et al.^[^ ^127^ ^]^ endowed THs with antifreeze properties by incorporating LiCl or EG to the as‐prepared hydrogel electrolytes, achieving feasible energy harvesting under extreme temperatures. However, other factors that affect TH stability still remain unattended—performance degradation in quasi‐solid‐state TECs over time can result from water evaporation, chemical breakdown of the polymer matrix, and redox couple aggregation, all of which can significantly impact the reliability and efficiency of TECs in practical applications. Addressing these problems can effectively promote the development of THs.
- Understanding component interactions: Gaining deeper insights into the interactions between polymers, ions, and solvents are crucial. These interactions play a critical role in determining the hydrogel microstructures, cation/anion diffusion, and redox reactions, all of which impact the thermoelectrochemical performance. Alongside experimental studies, computational methods, such as MD simulations and first‐principle calculations like Ab Initio MD and DFT should be employed. These approaches enable a direct examination of interactions between components and potential chemical/electrochemical reactions within THs. Furthermore, microscopic phenomena such as ion solvation and transport, which were routinely assessed through computational approaches, link component interaction to continuum‐scale Seebeck coefficient and electrical conductivity. Insights into the relationship between component interaction, microscopic phenomena, and continuum‐scale properties of THs will illuminate pathways for designing enhanced THs.
- High‐throughput design: By integrating computational tools such as MD, AIMD, DFT, with machine learning (ML) algorithms, vast chemical and structural spaces can be screened rapidly to identify optimal combinations of polymers, redox couples, and additives. These approaches facilitate the prediction of key properties, such as ionic conductivity and Seebeck coefficient, while minimizing the cost associated with extensive experimental trials. Coupled with ML‐driven data analysis, high‐throughput computational approaches can uncover intricate structure‐property relationships and identify molecular descriptors to guide the rational design of THs. Although this framework has been successfully applied to the predictive design of electrolytes for energy storage, its implementation in TH research faces unique challenges. Currently, experimentally available datasets for key parameters, such as thermopower, are scarce, which restricts the training and validation of computational models. Moreover, there is a need for highly efficient computational approaches to address the complexities inherent in TH systems. For instance, as discussed in Section 4.2, the FEP in MD simulation was employed to predict the solvation entropy difference of redox couples. However, this method requires extensive sampling of free energies at multiple temperatures, making it computationally impracticable for high‐throughput calculations. Overcoming these challenges will require advancements in efficient algorithms to quantify thermopower. Additionally, the development of robust datasets and the integration of multi‐scale modeling techniques are necessary to bridge atomic‐level interactions with macroscopic device performance.
- Device optimization and practical applications: While TH‐based devices show promising performance in laboratory setting, a significant gap remains between experimental development and practical application. To bridge this gap, research must prioritize high‐efficiency device design, low‐cost material development, scalability, innovative cell structures/operation modes, and material selection for diverse application scenarios. In practical applications, different device structures may be employed based on practical demands and material characteristics, thereby enhancing energy conversion efficiency under various conditions. In addition, creating p‐n series circuits is essential for device design. Establishing low‐cost manufacturing methods (e.g., 3D printing, spray coating, etc.) can facilitate large‐scale fabrication and commercialization. For example, Yan et al.^[^ ^112^ ^]^ developed integrated TH‐based devices spanning 2.3 m in length that could generate an output voltage of ≈45 V through a unique combination of series‐parallel integration. Furthermore, Niu et al.^[^ ^104^ ^]^ reported a TH‐based device made up of nine units that can generate a voltage of 1.76 V using outdoor photothermal heat. In another work, by 3D printing, a large‐scale and flexible TH‐based module consisting of 36 units was created, which generated a voltage of 0.58 V from body heat (ΔT ≈5 K).^[^ ^65^ ^]^ These emerging manufacturing technologies not only offer cost‐effective solutions but also provide precise control over the production process, which enables customizable and scalable TH fabrication in diverse forms. Furthermore, they simplify the creation of compact, highly integrated TH‐based array devices, thereby optimizing the use of low‐grade waste heat. Moreover, thermal management must be considered when designing devices to maintain the temperature difference between the electrodes. For example, in applications that harvest waste heat from the human body, it is crucial to ensure close contact between the device and skin to maximize thermal efficiency and maintain a consistent temperature on the air‐facing side. Unlike commercialized electronic thermoelectric materials (e.g., Bi2Te3), TH‐based devices require further optimization to meet practical application needs effectively.
- High‐performance electrode design: Electrode materials play a critical role in the quasi‐solid‐state TECs, as they directly influence the interfacial dynamics and the overall heat‐to‐electricity conversion efficiency of THs. To optimize electrode performance, efforts should concentrate on the following key aspects: 1) Modifying the electrolyte/electrode interface to enhance ion transport (e.g., minimizing contact resistance, introducing beneficial function groups); 2) Developing new electrodes such as MXene, conductive polymers, and graphene‐based materials, which offer high electrical conductivity, tunable surface chemistry, and excellent compatibility with THs to improve TECs efficiency; 3) Conducting systematic investigation of the impact of electrode geometry, including size, thickness, and configuration on heat‐to‐electricity conversion efficiency. Advancing thermoelectric technology requires a thorough understanding of how electrode properties influence overall device performance.

## Conflict of Interest

The authors declare no conflict of interest.
